# Suppression of Cofilin function in the somatosensory cortex alters social contact behavior in the BTBR mouse inbred line

**DOI:** 10.1093/cercor/bhae136

**Published:** 2024-04-10

**Authors:** Iris W Riemersma, Kevin G O Ike, Thomas Sollie, Elroy L Meijer, Robbert Havekes, Martien J H Kas

**Affiliations:** Groningen Institute for Evolutionary Life Sciences, Neurobiology, University of Groningen , Nijenborgh 7, 9747 AG Groningen, The Netherlands; Groningen Institute for Evolutionary Life Sciences, Neurobiology, University of Groningen , Nijenborgh 7, 9747 AG Groningen, The Netherlands; Groningen Institute for Evolutionary Life Sciences, Neurobiology, University of Groningen , Nijenborgh 7, 9747 AG Groningen, The Netherlands; Groningen Institute for Evolutionary Life Sciences, Neurobiology, University of Groningen , Nijenborgh 7, 9747 AG Groningen, The Netherlands; Groningen Institute for Evolutionary Life Sciences, Neurobiology, University of Groningen , Nijenborgh 7, 9747 AG Groningen, The Netherlands; Groningen Institute for Evolutionary Life Sciences, Neurobiology, University of Groningen , Nijenborgh 7, 9747 AG Groningen, The Netherlands

**Keywords:** animal models, autism spectrum disorder, social behavior, structural plasticity, synaptic plasticity

## Abstract

Sensory differences are a core feature of autism spectrum disorders (ASD) and are predictive of other ASD core symptoms such as social difficulties. However, the neurobiological substrate underlying the functional relationship between sensory and social functioning is poorly understood. Here, we examined whether misregulation of structural plasticity in the somatosensory cortex modulates aberrant social functioning in BTBR mice, a mouse model for autism spectrum disorder–like phenotypes. By locally expressing a dominant-negative form of Cofilin (Cofilin^S3D^; a key regulator of synaptic structure) in the somatosensory cortex, we tested whether somatosensory suppression of Cofilin activity alters social functioning in BTBR mice. Somatosensory Cofilin suppression altered social contact and nest-hide behavior of BTBR mice in a social colony, assessed for seven consecutive days. Subsequent behavioral testing revealed that altered social functioning is related to altered tactile sensory perception; Cofilin^S3D^-treated BTBR mice showed a time-dependent difference in the sensory bedding preference task. These findings show that Cofilin suppression in the somatosensory cortex alters social functioning in BTBR mice and that this is associated with tactile sensory processing, a critical indicator of somatosensory functioning.

## Introduction

Autism spectrum disorder (ASD) is a complex neurodevelopmental condition characterized by differences in social interaction, restricted or repetitive behaviors, and sensory differences that begin early in life. Sensory differences are reported in up to 90% of the individuals diagnosed with ASD of which somatosensory hyper- or hyporeactivity is specifically common among 60% of the autistic people (Kientz and Dunn 1997; Rogers and Ozonoff 2005; Tomchek and Dunn 2007; Puts et al. 2014). Sensory symptoms in general are one of the earliest predictors and indicators of ASD. Although diagnoses generally take place within the first 3 years of life, sensory symptoms can be found as early as 6 months of age (Estes et al. 2015). In addition, sensory symptoms highly correlate with other core symptoms such as social interaction (Wiggins et al. 2009; Tavassoli et al. 2014; Miguel et al. 2017; Kirby et al. 2022). Growing evidence suggests that sensory symptoms, in particular tactile symptoms, contribute to the social differences in ASD (Schaffler et al. 2019). However, which neurobiological mechanisms contribute to these sensory abnormalities in ASD is still poorly understood.

The somatosensory cortex processes tactile information from somatic sensations. These sensations include touch, pressure, vibration, proprioception (i.e. the position of the body in space), nociception (i.e. pain), and temperature. In addition, the somatosensory cortex plays a role in higher-order cognitive functions such as tactile attention, emotion, and empathy (Kropf et al. 2019). For these reasons, it is also considered essential for social behavior. Work by the Ginty lab indicates that targeting the peripheral somatosensory neurons in ASD mouse models can alleviate ASD behaviors, including sensory behavior and forms of social behavior (Orefice et al. 2016; Orefice et al. 2019). These observations underscore the potential involvement of the somatosensory system in both social behavior and ASD. However, the question remains whether a direct relationship exists between central nervous system (CNS) higher-order sensory processes, specifically in the somatosensory cortex, and social functioning in ASD and how this relationship is established.

Given the adaptive nature of the expression of sensory and social behavior, and considering synaptic dysfunction as a potential convergent biological mechanism underlying ASD, we hypothesized synaptic adaptation mechanisms as a candidate neurobiological target for investigating the relationship between sensory processes and social functioning (Benger et al. 2018; Guang et al. 2018; Riemersma et al. 2022). A candidate neurobiological substrate that is involved in synaptic plasticity is Cofilin. Cofilin is an actin-binding protein that regulates actin dynamics by disassembling actin filaments in the synapses. The cytoskeleton of dendritic spines is made up of these actin filaments that are essential for synapse structure and plasticity (Van Troys et al. 2008; Bernstein and Bamburg 2010; Bamburg et al. 2021). Active (nonphosphorylated) Cofilin can bind to actin and depolymerize actin filaments, whereas inactive (phosphorylated) Cofilin is unable to bind and disassemble actin filaments. Therefore, a balance between active and inactive Cofilin is essential for synaptic plasticity and spine stability. In addition, through regulating actin dynamics, Cofilin also regulates the presence of receptors in the synapse (Ackermann and Matus 2003; Gu et al. 2010; Duffney et al. 2015). BTBR T + tf/J (BTBR) mice, an inbred strain that shows ASD-like behavioral phenotypes, show disturbed maturation of dendritic spines at postnatal day 14, which could indicate differences in actin regulation (Moy et al. 2007; McFarlane et al. 2008; Silverman et al. 2010; Wei et al. 2015; Peleh et al. 2020; Xiao et al. 2020). Altered phosphorylation levels of Cofilin have been reported in genetic mouse models of ASD: *Shank3*- as well as *Nlg1*-deficient mice show increased Cofilin activity (Duffney et al. 2015; Liu et al. 2016). Moreover, Duffney et al. showed that inhibiting Cofilin function in the prefrontal cortex of *Shank3* mice ameliorated social deficits. These findings indicate a causal link between actin misregulation and ASD-linked phenotypes (Duffney et al. 2015). Despite these observations, to date, no studies have examined whether alterations in Cofilin function in the somatosensory cortex contribute to social phenotypes.

Here, we studied whether BTBR mice show strain differences in somatosensory Cofilin levels and whether such changes contribute to altered social functioning. We observed a decrease of phosphorylated Cofilin, but no changes in total Cofilin levels, in the somatosensory cortex of the BTBR mice, when compared to C57BL/6J mice that express higher levels of social contacts than BTBR mice. By means of viral vector–directed Cofilin intervention in the somatosensory cortex of these mice, we tested whether inhibition of increased Cofilin activity changes longitudinally impacted social functioning in this mouse strain in a seminatural social colony environment.

## Methods and materials

### Animals

Adult C57BL/6J and BTBR male mice were used in this study. For the BTBR and C57BL/6J comparison study, inbred male C57BL/6J (JAX stock #000664) and BTBR T+ Itpr3tf/J (JAX stock #002282) mice were ordered from The Jackson Laboratory (Bar Harbor, Maine, USA) via Charles River Europe (Den Bosch, The Netherlands). After arrival, mice, aged between 10 and 11 weeks, were housed in groups of 2 to 4 in Makrolon type 2 L cages for a minimum of 2 weeks before the start of the experiments. At the start of the experiment, all mice were 12 to 20 weeks of age (adult).

For the BTBR Cofilin intervention experiment, BTBR mice were bred in the animal facilities of the University of Groningen and originated from Jackson Laboratory (Bar Harbor, Maine, USA). At the start of the behavioral experiments, all mice were 15 to 22 weeks of age (adult). Mice were housed in groups of 2 to 4 in Makrolon-type 2 L cages. All mice were maintained under a 12:12 light/dark cycle, controlled temperature (21 ± 2 °C), humidity (55 ± 5%) and with ad libitum access to food and water. All animals were kept on standard bedding (Aspen) and had access to shredded cardboard nesting material, a cardboard tube, and a red plastic igloo as enrichment. All animals were randomized during testing and behavioral testing was performed by a researcher blinded to the conditions. All behavioral tests, except for the longitudinal recordings, took place during the dark phase. All experiments were performed in accordance with the European Communitives Council Directive (2010/63/EU) and according to the local rules set by the ethical authorities.

### Viral constructs

Cofilins’ ability to bind and depolymerize actin filaments is inhibited by phosphorylation at Serine 3 (Bamburg 1999). To alter synaptic plasticity in the somatosensory cortex, we used a phosphomimetic form of Cofilin, Cofilin^S3D^, in which serine at position 3 is substituted by aspartate as described previously (Havekes et al. 2016b). Consequently, similar to phosphorylation, S3D substitution of Cofilin inhibits its actin-binding capacity and actin-severing ability. More specifically, an adeno-associated virus (AAV) was used to overexpress inactive Cofilin (Cofilin^S3D^) in excitatory neurons of the somatosensory cortex of the mice. This was previously successfully used to inhibit Cofilin function (Havekes et al. 2016b; Vogel-Ciernia et al. 2017). An enhanced green fluorescent protein (eGFP) AAV served as a control. A CaMKIIα (calcium/calmodulin-dependent protein kinase II alfa) promoter was used to restrict the expression of the viruses to excitatory neurons (Havekes et al. 2016b). An HA-tag was added to the viral construct of Cofilin^S3D^ to be able to discriminate between endogenous Cofilin and viral exogenous Cofilin (Havekes et al. 2016b). The AAV5.CaMKII0.4.mCofilin.S3D-HA.rBG (Cofilin^S3D^) virus was constructed by the Vector Core of the University of Pennsylvania (stock titer: 1.07 × 10^13^ genome copies/mL). Mice were either injected with this AAV5.CaMKII0.4.mCofilin.S3D-HA.rBG or a control virus under the same promoter, a pAAV5-CaMKIIa-EGFP (eGFP) vector (Addgene, USA) (stock titer: 4.30 × 10^12^ genome copies/mL).

### Viral surgeries

Before the start of the surgery, the mice were anesthetized using isoflurane (5% induction, 1.8% maintenance) and a systemic injection of Carprofen (4 to 5 mg/kg). Mice were placed on a heating pad throughout the surgery. Lidocaine was locally administered on the skull as analgesia, and artificial tears (Duratears Z; Alcon) were used to protect the eyes from drying out. Mice were placed in a stereotax, a small incision was made, and holes were drilled into the skull above the somatosensory cortex of both hemispheres using a microdrill (Foredom). A 33G beveled needle (WPI) attached to a 10 μL Hamilton syringe was slowly lowered to the injection coordinates (A/P −1 mm, M/L+ and −2.9 mm, D/V −0.7 mm). After 1 min at −0.7 mm, the needle raised to −0.6 mm, and the virus was injected (0.3 μL; 0.1 μL/min). To control precision and speed, a micro syringe pump (UMP3, IWP) was connected to a stereotaxic frame and controller (Micro4, IWP). Either the Cofilin^S3D^ or eGFP AAV virus was injected bilaterally. A minute after injection, the needle was slowly removed. After surgery, the skin was glued. During recovery from anesthesia, the mice were placed in a cage on a heating mat (5 to 10 min). After recovery from surgery, the mice were group-housed with their original cage mates in mixed Cofilin^S3D^ and eGFP-treated cages. The viral surgeries were performed at 11 weeks of age, behavioral experiments were performed after 4 weeks to allow for optimal expression of the virus.

### Radio-frequency identification chip implantation

One week prior to the start of the BARISTA experiment mice were injected with a radio-frequency identification (RFID) chip (12 mm long, 2.12 mm diameter) to locate their position and movement in the arena. Injection of the chip took place under anesthesia (i.e. isoflurane ∼2%). First, the skin around the implantation site was cleaned with chlorhexidine alcohol. Next, using a specialized syringe, the RFID chip was injected subcutaneously into the dorsal/caudal flank of the mouse to ensure an upright position of the chip under the skin.

### Behavioral Automated RFID-Integrated Social Tracking Arena longitudinal behavioral assessment

**Fig. 1:**
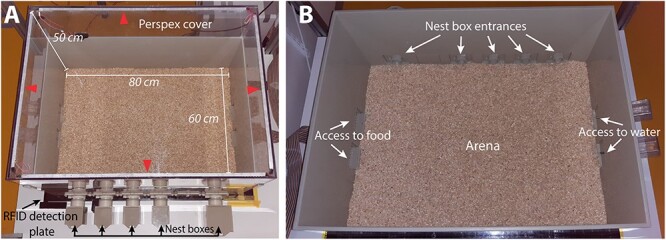
The BARISTA system. A) The BARISTA with bedding on top of the RFID plate (black plate at the bottom) and the translucent cover on top of the arena. The white and infrared LED strips (arrowheads) attached along the sides of the cover ensure equal illumination of the open space. Four smaller nest boxes and one bigger nest box are shown at the bottom of the picture. B) The BARISTA without the cover, showing the entrances of the nest boxes at the top of the picture and the food hoppers and the water bottles on the left and right side, respectively (adapted from Lanooij et al. 2023).

Individual social behaviors in group-housed mice were monitored in the Behavioral Automated RFID-Integrated Social Tracking Arena (BARISTA) system. The BARISTA system is a seminatural environment in which mice were monitored for seven consecutive days (Peleh et al. 2019; Peleh et al. 2020; Lanooij et al. 2023). The mice were housed in groups of four mice per colony, for the strain phenotyping, four BTBR and four C57BL/6J mice per arena were used with a total of three colonies per strain. For the Cofilin intervention experiment, four BTBR-eGFP and four BTBR- Cofilin^S3D^ mice per arena were used with a total of four colonies per strain with the exception of one BTBR-eGFP colony consisting of three mice. On the first experimental day, the mice were placed in the arena. The mice placed together in one arena originated from different cages and had not interacted before the start of the experiment. The social tracking arena consisted of one open arena (80 × 60 × 50 cm) filled with approximately 1 to 2 cm of standard local bedding material, four small nest boxes (7 × 7 × 7 cm), and one big nest box (10 × 10 × 10 cm) that connect to the open arena through short tunnels (∅ 4 × 7 cm) (Fig. 1). The open arena and nest boxes were made of opaque PVC, and the open arena was covered with a translucent Perspex cover. White and red light-emitting diodes (LEDs) were attached to all sides of the cover to ensure equal illumination of the open arena. Mice were maintained under a 12:12 light/dark cycle, controlled temperature (21 ± 1 °C) and humidity (55 ± 5%), and with ad libitum access to food and water. Mice were left undisturbed during the 7-day recording except for short animal welfare checks.

Behavioral characterization of social behaviors was performed using a combination of video and RFID signals utilizing the RFID-assisted Social Scan software (Clever Sys Inc., Reston, Virginia, USA) (Peleh et al. 2019; Peleh et al. 2020). The video was recorded using a Basler acA1300-60gmNIR GigE monochrome camera equipped with an IR pass filter (850 nm), while an RFID plate containing 24 RFID coils under the open arena registered the RFID chips. Behaviors in the open arena were automatically assessed with Social Scan based on the RFID-supported video tracking, resulting in unbiased and consistent scoring. Detection settings for the behavioral characterization included parameters such as moving direction (angle), distance between animals, and distance moved for automated behavioral analysis of social contact, social approaching, social sniffing, social following, social leaving, and nest hiding (Table 1). Nest hide is defined as a mouse spending time hiding in the nest boxes. Time spent in and around the nests is defined as a mouse spending time either hiding in the nest boxes or being located just in front of the nest boxes, on the side of the open arena.

**Table 1:** Detector settings of social scan used for automated behavioral scoring. Social contact is measured based on the physical contact of a mouse with one or more conspecifics. Social sniffing refers to all sniffing of a conspecific at any location of the body. Approach, leave, and follow behaviors refer to movement of one mouse toward or away from a conspecific. All these behaviors were only measured in the open arena and not in the nest boxes (Lanooij et al. 2023).

**Social contact**	
Distance between mice	2 cm
Minimum duration	0.5 s
**Social sniffing**	
Distance between mice	<3.5 cm
Moving direction (angle) of mouse 1	<45°
Minimum duration	0.33 s
**Approach**	
Distance between mice	<100 cm
Moving direction (angle) of mouse 1	<45°
Distance to be traveled by mouse 1 towards mouse 2	>7 cm
Velocity of mouse 1	>4 cm/s
**Social leave**	
Distance between mice	<100 cm
Moving direction (angle) of mouse 1	<45°
Distance to be traveled by mouse 1 away from mouse 2	>14 cm
Velocity of mouse 1	>4 cm/s
**Social follow**	
Distance between mice	<30 cm
Moving direction (angle) of mouse 1	<45°
Moving direction (angle) of mouse 2	<90°
Distance to be traveled by mouse 1 and 2	>7 cm
Velocity of mice	>4 cm/s

### The social preference test

This test is based on the premise that rodents prefer social over nonsocial stimuli and is commonly used to assess social preference or social avoidance behaviors. This test was performed in a three-compartment cage (i.e. the so-called three-chamber apparatus). Mice were habituated to the experimental room for 30 min before the start of the experiment. At the start of the test, the mouse was habituated in the middle compartment for 10 min without access to the other compartments. Subsequently, the mouse was allowed to explore all three compartments for another 10 min. During this exploration, one chamber contains a nonsocial stimulus (empty cage), and one chamber contains a social stimulus (unfamiliar encaged stimulus mouse). The stimulus mice were unfamiliar age- and sex-matched A/J mice that were randomly placed in one of the wired cages in the outer chambers. Social preferences/avoidance was assessed by analysis of the time spent exploring the social and nonsocial stimulus and chamber using Ethovision XT 11.5 (Noldus Information Technology BV, Wageningen, the Netherlands).

### Social discrimination task

Both short- and long-term social recognition was assessed in the social discrimination task. Mice were habituated to the experimental room for 30 min. After habituation to the room, the subject mouse was placed in a standard transparent housing cage (Makrolon type 2 L) with bedding for 5 min to habituate to the experimental environment. After habituation, during the learning phase, the subject mouse was exposed to an unfamiliar male stimulus mouse of the same age for 2 min to familiarize itself. Thereafter, the stimulus mouse was removed, followed by an inter-trial interval of 5 min. Subsequently, during the short-term testing phase, the familiar mouse and a novel mouse (novel 1) were put in the cage with the subject mouse for another 2 min to test short-term recognition. The long-term testing phase took place 24 h after the learning phase. The test was carried out with the familiar mouse and a second novel mouse (novel 2). Before the long-term testing phase, the subject mouse habituated for 5 min to the testing environment. The trials were recorded, and social interaction with conspecifics was scored using The Observer XT 14 (Noldus Information Technology BV, Wageningen, the Netherlands).

### Repetitive behavior

To assess for repetitive behavior mice were first recorded for an hour in a normal housing cage (Makrolon type 2 L) with bedding and subsequently for 10 min in a housing cage without bedding. Four small objects (lego block, pion, dice, and marble) were put in the corners of the cage with bedding to stimulate exploration behavior. In the first 10 min of the recording, the following behavior was scored: rearing, jumping, grooming (in trials without bedding), and digging (in trials with bedding) using the Observer XT 14 software (Noldus Information Technology BV, Wageningen, the Netherlands). In addition, distance moved was tracked using the Ethovision XT 11.5 software (Noldus Information Technology BV, Wageningen, the Netherlands). Lastly, in the hour-long trials, repetitive locomotion patterns were analyzed as previously described (Bonasera et al. 2008; Molenhuis et al. 2018). In short, the arena was divided into nine areas where the mouse spent an equal amount of time. Subsequently, the Theme software version 5.0 (Noldus Information Technology BV, Wageningen, the Netherlands) was used to detect patterns of locomotor activity and to calculate the total percentage of time a mouse spent in patterns.

### Buried food test

The buried food test was carried out to assess general olfactory ability (Yang et al. 2012). Prior to the test, the subject mouse was food-deprived for 18 h. The test was performed in a standard transparent housing cage (Makrolon type 2 L) containing 3 cm of clean bedding material. Mice were allowed to habituate for 5 min; subsequently, the mouse was briefly removed from the cage and a small food pellet (1 cm^3^) was buried approximately 1 cm underneath the bedding in one of the corners. The subject mouse was placed back into the cage and the latency to dig and uncover the food pellet as well as the latency to eat the food pallet was scored using The Observer XT 14 software (Noldus Information Technology BV, Wageningen, the Netherlands).

### Bedding preference test

The bedding preference test was performed to measure sensory preference when exposed to two types of bedding. This test was based on the social conditioned place preference test and was performed in a three-chamber apparatus (Pearson et al. 2012). The outer compartments were filled with a layer of bedding material, while the middle compartment remained empty. The mice were exposed to two types of bedding, on one side corncob bedding (J. Rettenmaier & Söhne) and on the other side paper chip bedding (Shepherd). At the start of the experiment, the mouse was placed in the middle compartment. Subsequently, the doors to the outer compartments were opened and the subject mouse was allowed to explore the chambers freely for 30 min. The preference was determined by measuring how much time was spent in each compartment using the Ethovision XT 11.5 software (Noldus Information Technology BV, Wageningen, the Netherlands).

### Western blotting

Animals were sacrificed by cervical dislocation and whole brains were snap-frozen in 2-methylbutane on dry ice. Subsequently, brains were sliced in 100 μm thickness coronal sections in a cryostat (Slee MNT) at −13 °C. Brain sections were then mounted on microscope slides, and the somatosensory cortex was collected bilaterally via 0.8 mm punches using hollow needles. Tissue was stored at −80 °C until further processing. Protein extraction from the sample was done using Tissue-lyser (Quiagen, Germany) and homogenizer beads. Samples were lysated in Coba buffer (Tris, Na- Deoxycholate (DOC), NaF, sodium orthovanadate, ethylenediaminetetraacetic acid (EDTA), and sodium β-glyc, PhosStop X1, Complete Mini). The latter two molecules are phospho- and protease inhibitors and were added freshly prior to lysis. After lysation, samples were centrifuged at 13,000 rpm at 4 °C, and supernatant was collected. Protein levels were determined and normalized using a Lowry-based Bio-rad DC protein assay. Final protein concentrations were normalized within each group by using additional lysis buffer, and LDS sample buffer was added at one-fourth of the total volume. Subsequently, 25 μg protein was run onto a pre-cast BoltTM 4 to 12% Bis-Tris Plus (Invitrogen, USA) gel. After electrophoresis, the samples were transferred from gel to membrane with iBlotTM Gel Transfer System (Invitrogen, USA). Next, the membranes were blocked for 1 h with 5% BSA or 5% skim milk and incubated overnight with either one of the following primary antibodies: anti-p-Cofilin (1:750, Cell Signaling Technology, RRID: AB_2080597), anti-Cofilin (1:2,000, BD Biosciences, RRID: AB_399516), HA-Tag (1:1,000, Cell Signaling Technology, RRID: AB_10691311), and Glyceraldehyde 3-phosphate dehydrogenase (GAPDH) (1:3,000, Thermo Fisher Scientific, RRID: AB_568547). After incubation with the primary antibodies, the membranes were incubated with the corresponding HRP-conjugated antibodies mouse secondary antibody (GAPDH 1:6,000; rest 1:5,000, Thermo Fisher Scientific, RRID: AB_228307) and rabbit secondary antibody (1:5,000, Cell Signaling, RRID: AB_2099233) for 2 h at room temperature. Bands were visualized by first incubation with ECL solution (ThermoFisher Scientific, USA) and subsequent imaging using the Molecular Imager ChemiDoctm XRS System (BioRad, USA).

### Immunochemistry

Mice were perfused by transcardial perfusion and brains were collected for immunohistochemistry to determine whether the virus was injected in the correct location. Mice were first perfused with 0.9% NaCl and 2 U/mL heparin followed by fixation solution containing 4% PFA in 0.1 M PB, after which the brains were post-fixated in the fixative for 24 h at 4 °C. After dehydration with 10%, 20%, and 30% sucrose, brains were frozen and subsequently sliced in 20 μm thick coronal sections in a cryostat (Leica LM 3050) at −14 °C. Sections were then washed withphosphate-buffered saline (PBS) and then incubated with 0.3% H_2_O_2_. Subsequently, sections were blocked with 3% normal goat serum (NGS) and 0.1% TritonTM X-100 in PBS. Then, sections were incubated with the following antibodies: HA-Tag (1:200, Cell Signaling Technology, AB_10691311) and corresponding Alexa fluor-conjugated secondary antibodies (1:00, Invitrogen). Sections were then mounted with VECTASHIELD Vibrance Antifade Mounting Medium with DAPI (Vector Labs, United Kingdom). Fluorescent images were taken using a Leica confocal microscope. In 4 out of the 15 mice used for immunohistochemistry, only one hemisphere showed clear staining for the virus (3 Cofilin-S3D- and 1 eGFP-injected mouse); in 1 eGFP-injected mouse, no autofluorescence could be observed. In addition, in two eGFP-injected mice, viral spread to parts of the hippocampus was seen; however, in none of the Cofilin-S3D mice, this was observed. Therefore, none of the mice were excluded from the study.

### Data analysis

Analysis of the BARISTA group housing data was performed by means of generalized additive mixed modeling (GAMM) in RStudio (version 2022 July 1), making use of the packages *plyr*, *nlme*, *mgcv*, *itsadug*, *ggplot2*, *extrafont,* and *cowplot* (Wood 2003; Wood 2004; Wickham 2011; Wood 2011; van Rij et al. 2016; Wilke 2016; Wood et al. 2016; Wickham 2017; Wood 2017; R Foundation for Statistical Computing 2018; Wickham et al. 2019; Pinheiro et al. 2021). GAMM is an extended version of linear mixed-effects modeling and was chosen over linear modeling because the relation between the outcome variables and time is cyclic rather than linear in the longitudinal recording of social behavior in the BARISTA system (Sala and Galuzzi 2023; Wieling 2018; Mundo et al. 2022). Data on the social colony behaviors were collected as cumulative frequency and/or duration of time spent on the behavior in the respective arena in bins of 1 h. Separate models were built for the outcome measures; with AIC-based model comparison, the best models were determined. An overview of the final models can be found in the supplementary materials (Supplementary Table 1 for the BTBR C57BL/6J comparison and Supplementary Table 2 for the BTBR-Cofilin^S3D^ and BTBR-eGFP comparisons). Data were transformed when applicable and then modeled based on a Gaussian or quasipoisson distribution. The statistical significance of the effect of the intervention was assessed over time using GAMM models for repeated measures. This was followed by the inspection of difference plots at specific time points between the groups (C57BL/6J vs. BTBR and BTBR-Cofilin^S3D^ vs. BTBR-eGFP) of which the C57BL/6J and BTBR-eGFP were used as the respective reference groups. The time points or intervals where the confidence intervals (CI) of the pairwise group comparison did not cover zero were considered significant (Mundo et al. 2022).

In addition, since the different outcome measures were highly correlated at specific time points, we assessed differences between Cofilin^S3D^- and eGFP-treated mice over the total inactive and active phases of the recording. For this analysis, the cumulative frequency or duration of time spent on the behavior in their respective arena in the active or inactive phases was analyzed and tested for significance using either a *t*-test or a nonparametric alternative with appropriate correction for multiple comparisons. Figures regarding the BARISTA data represent the predicted data over week recording based on these models as well as showing the raw data over the mean 24 h and for the active and inactive phases. Data from other behavioral paradigms were assessed using *t*-tests, or if the criteria for normality were not met based on the Shapiro–Wilk test, it was assessed with the use of a Mann–Whitney U test. Data were corrected for multiple testing using when applicable using the FDR method.

## Results

### Decreased social contact, but increased nest hide behavior in BTBR mice

We first examined the strain differences in social behavior between BTBR and C57BL/6J mice. To this end, individual social behaviors in group-housed BTBR mice and C57BL/6J controls were monitored for a week in the BARISTA system (Fig. 1 and Table 1). This data set was previously analyzed to assess different behavioral outcome measures (Peleh et al. 2020).

**Fig. 2:**
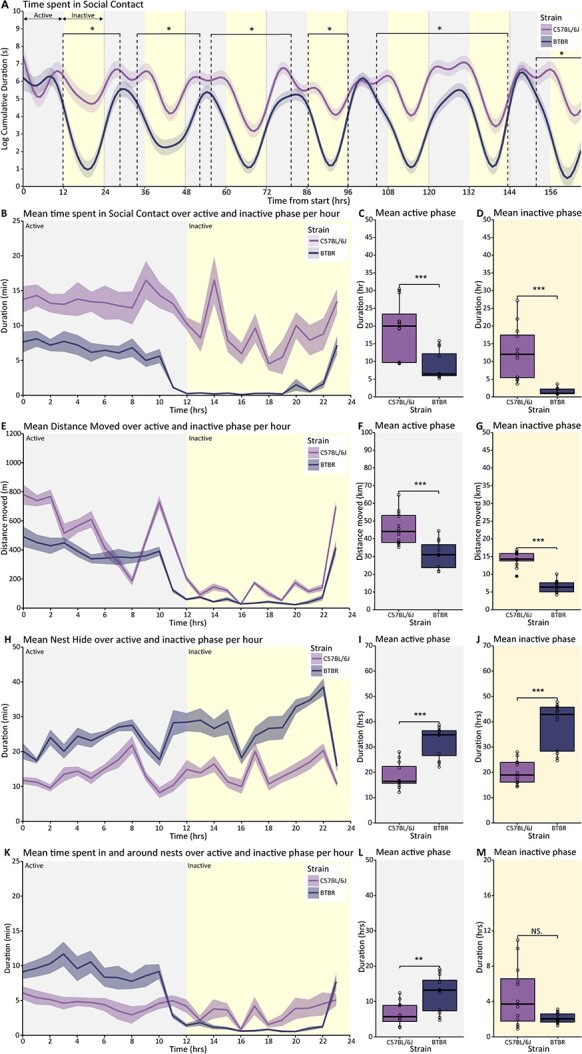
Strain differences in social and locomotor behaviors between BTBR and C57BL/6J mice. A) Predicted time spent in social contact during the 7 days of recording in the BARISTA system based on modeled data. Data are presented as the logarithm of cumulative duration (mean ± SEM) based on 1 h bins, with time from the start of the experiment on the *x*-axis in hours. B) Mean time spent in social contact over the active and inactive phase of the recording averaged per hour of the active and inactive phase. Mean time spent in social contact during the active C) and inactive D) phases over the recording period. E) Mean distance moved over the active and inactive phase of the recording averaged per hour of the active and inactive phase. Mean distance moved during the active F) and inactive G) phase over the recording period. H) Mean time spent hiding in the nests over the active and inactive phase of the recording averaged per hour of the active and inactive phase. Mean time spent hiding in the nests during the active I) and inactive J) phase over the recording period. K) Mean time spent in and around the nests over the active and inactive phase of the recording averaged per hour of the active and inactive phase. Mean time spent in and around the nests during the active L) and inactive M) phase over the recording period. BTBR animals (*n* = 12) are shown in dark purple, and C57BL/6J controls (*n* = 12) are shown in light colored lines and bars. The 24 hours light-dark cycle with 12 hours of light (inactive phase) and 12 hours of darkness (active phase) are indicated in different shadings behind the graphs. Boxplot represents median and quartiles with minimum and maximum whiskers; line graph presents mean ± SEM. ^*^P < 0.05, ^**^P < 0.01, ^***^P < 0.001.

Since differences in motor activity level can highly impact other behavioral readouts, we first examined distance moved in the BARISTA. BTBR mice show decreased distance moved compared to C57BL/6J controls, consistent over both the active and the inactive phase (strain*time interaction *F*(55.882, 3556.426) = 10.28; *P* < 0.001; inactive phase *P* < 0.001, active phase *P* < 0.001; Fig. 2E–G, Supplementary Fig. 1A). To identify social behaviors that are not directly influenced by motor activity levels, correlation analyses were performed between distance moved and the social outcome measures from the BARISTA-system. This analysis revealed that distance moved is strongly correlated with social behaviors such as approach or follow, but not with other behaviors such as social contact or nest behaviors (Supplementary Fig. 2). For these reasons, social contact and nest behaviors were defined as the primary outcome measures for this study.

As a next step, we performed a strain comparison in social contact and nest-related behaviors in the BARISTA system between BTBR and C57BL/6J mice. BTBR mice expressed less social contacts compared to C57BL/6J controls (strain*time *F*_(53.222, 3,574.298)_ = 21.57; *P* < 0.001; Fig. 2A). Taking all days together, this was observed both in the inactive and active phases of the average 24 h period (*P* < 0.001; Fig. 2B–D). In contrast to this decrease in social contact, BTBR mice spent more time in the nest boxes than C57BL/6J mice during both the active and inactive phases (strain*time *F*_(61.774, 3,580.031)_ = 4.62; *P* < 0.001; *P* < 0.001; Fig. 2H–J, Supplementary Fig. 1B). Similarly to the increased nest hide, there was also a significant interaction between strain and time in the amount of time mice spent both in the nest boxes or around the area of the nest boxes (strain*time *F*_(56.733, 3,549.093)_ = 13.44; *P* < 0.001; Supplementary Fig. 1C). Further assessment of the overall active and inactive phases shows that BTBR mice spent more time in and around the nest boxes during the active phase specifically (active phase *P* < 0.01, Fig. 2K–M). Overall, BTBR mice spent less time in social contact and spent more time in and around the nest boxes.

### Decreased levels of inactive Cofilin in the somatosensory cortex of BTBR mice

**Fig. 3:**
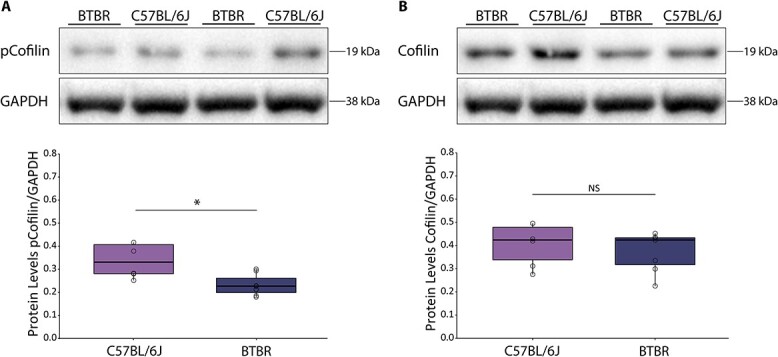
Decreased levels of inactive Cofilin in the BTBR mice compared to C57BL/6J. A) Western blot analysis of phosphorylated Cofilin (pCofilin) protein levels in the somatosensory cortex. GAPDH was used as an internal control. A representative blot is shown. Each band represents an individual animal (*P* = 0.047; C57BL/6J *n* = 5; BTBR *n* = 7). B) Western blot analysis of total Cofilin (Cofilin) protein levels in the somatosensory cortex. GAPDH was used as an internal control; a representative blot is shown. Each band represents an individual animal (*P* > 0.05; C57BL/6J *n* = 5; BTBR *n* = 7). Boxplot represents median and quartiles with minimum and maximum whiskers.

Next, we assessed whether BTBR mice show altered Cofilin regulation in the somatosensory cortex compared to C57BL/6J controls. Protein assessment of Cofilin levels in the somatosensory cortex showed a decrease in phosphorylated (inactive) Cofilin levels in the BTBR mice when compared to the C57BL/6J controls (Fig. 3A, *P* = 0.047), but no differences were found in total Cofilin levels (Fig. 3B, *P* > 0.05). These findings indicate that Cofilin function is misregulated in the somatosensory cortex of BTBR mice.

### Overexpression of Cofilin^S3D^ in the somatosensory cortex does not change overall locomotor activity in the BTBR mice

**Fig. 4:**
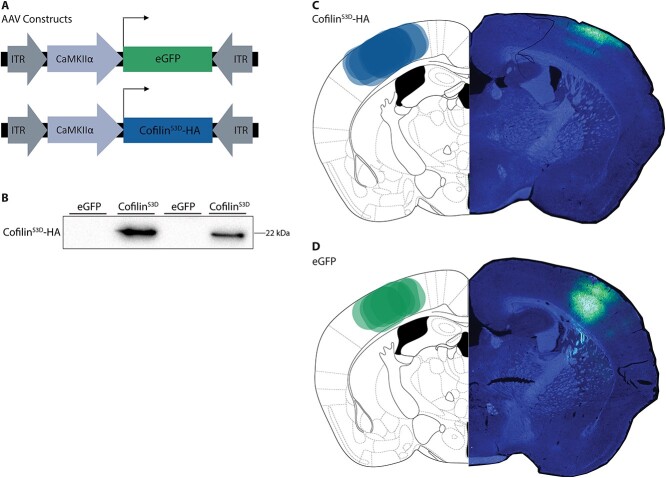
Viral suppression of Cofilin in the somatosensory cortex of BTBR mice. A) Mice were injected with pAAV5-CaMKIIα0.4-eGFP or pAAV5-CaMKIIα0.4-CofilinS3D-HA into the somatosensory cortex to drive expression of either eGFP, as a control, or the mutant inactive form of Cofilin (Cofilin^S3D^) in excitatory neurons. An HA-tag was included to discriminate between mutant and endogenous Cofilin. B) Western blot analysis of virally delivered Cofilin^S3D^ protein levels in the somatosensory cortex. An HA-tag antibody was used to detect the mutant inactive form of Cofilin (*n* = 2). C, D) Injection site of the AAVs into the somatosensory cortex (*n* = 8 for CofilinS3D and *n* = 7 for eGFP). Oval shaped areas in the left hemisphere (C + D) represent the overlapping virus injection sites in the somatosensory cortex for the tested animals. These areas represent the overlapping virus injection sites for the tested animals. Right hemisphere shows a representative staining of the virus in the somatosensory cortex. In general, autofluorescence of the eGFP proteins gives a stronger signal throughout the cortex, while antibody-based fluorescence of the HA-tag shows less staining in the deeper layers of the cortex.

Based on the observed differences in Cofilin phosphorylation levels, we wanted to determine whether inhibition of Cofilin activity can alter social functioning in the BTBR mouse strain. To this end, we expressed a dominant negative form of Cofilin (Cofilin^S3D^) (Havekes et al. 2016b; Vogel-Ciernia et al. 2017) in the somatosensory cortex of BTBR mice (Fig. 4A). Viral eGFP expression served as a control (Fig. 4A and D). Western blot analysis and immunohistochemistry confirmed that Cofilin^S3D^ was expressed in the somatosensory cortex (Fig. 4B and C). In additional Western blot analyses of Cofilin levels in the four groups (untreated C57BL6/J, BTBR, and treated BTBR-eGFP and BTBR-Cofilin^S3D^) revealed no differences between the four groups (Supplementary Fig. 3). This indicated that the inhibition of Cofilin levels by means of viral injection did not alter phosphorylated Cofilin in a supraphysiological manner.

**Fig. 5:**
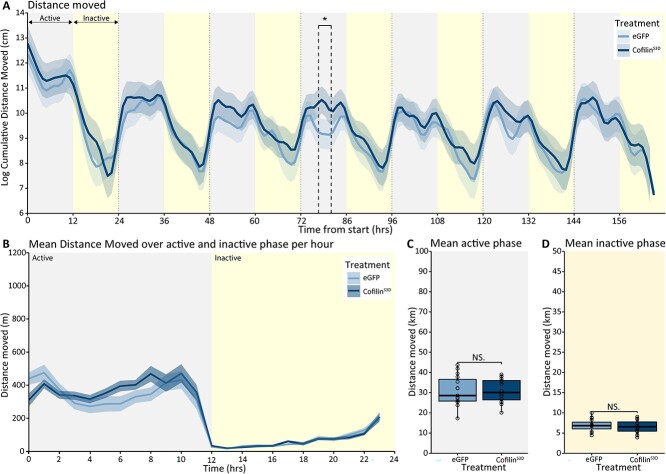
Distance moved in BTBR Cofilin^S3D^ and control eGFP mice. A) Predicted distance moved during the 7 days of recording in the BARISTA system based on modeled data. Data are presented as the logarithm of cumulative duration (mean ± SEM) based on 1 h bins, with time from the start of the experiment on the *x*-axis in hours. B) Mean distance moved over the active and inactive phase of the recording averaged per hour of the active and inactive phase. Mean distance moved during the active C) and inactive D) phases over the recording period. BTBR-Cofilin^S3D^ animals (*n* = 16) are shown in dark colored lines and bars, and BTBR controls (*n* = 15) are shown as light colored lines and bars. The 24 hours light-dark cycle with 12 hours of light (inactive phase) and 12 hours of darkness (active phase) are indicated in different shadings behind the graphs. Boxplot represents median and quartiles with minimum and maximum whiskers; line graph presents mean ± SEM. ^*^P < 0.05.

To investigate whether Cofilin^S3D^ expression in the somatosensory cortex affected social behavior, mice were monitored for seven consecutive days in the BARISTA system. First, we checked whether Cofilin suppression led to changes in motor activity levels. Overexpressing Cofilin^S3D^ did not lead to any general changes in locomotor activity (Fig. 5). It should be noted, however, that BTBR-Cofilin^S3D^-treated mice showed a significant increase in distance moved during a 3 h window during the active phase of the fourth day (treatment*time interaction *F*_(54.19, 4,861.50)_ = 16.47; *P* < 0.001: time window of significant difference 76.67 to 80, Fig. 5A). This effect was not consistent over other active phases or during any other time points. Moreover, the overall motor activity levels over the active or inactive phases were not significantly different (Fig. 5B–D), indicating that the brief difference in activity might be a result of natural variations in the circadian activity pattern (Tankersley et al. 2002; Brown et al. 2016). Therefore, we conclude no effect of Cofilin inhibition on overall locomotor activity.

### Overexpression of inactive Cofilin in the somatosensory cortex decreases social contact of BTBR mice during the active phase

**Fig. 6:**
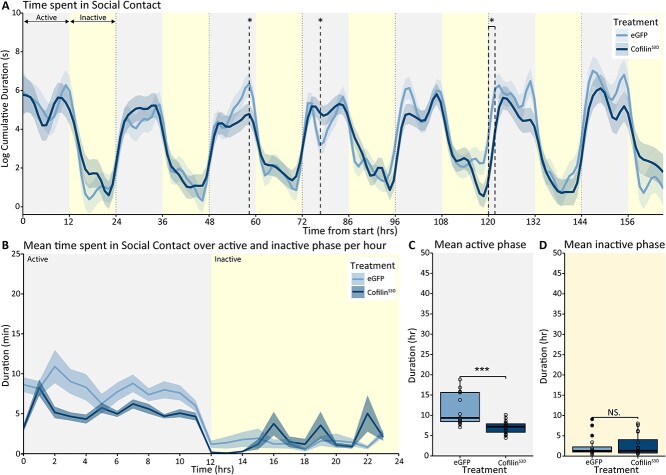
Social contact in BTBR Cofilin^S3D^ and control eGFP mice. Predicted time spent in social contact during the 7 days of recording based on modeled data. A) Data are presented as the logarithm of cumulative duration (mean ± SEM) based on 1 h bins, with time from the start of the experiment on the *x*-axis in hours. B) Mean time spent in social contact over the active and inactive phase of the recording averaged per hour of the active and inactive phase. Mean time spent in social contact during the active C) and inactive D) phases over the recording period. BTBR-Cofilin^S3D^ animals (*n* = 16) are shown in dark colored lines and bars, and BTBR controls (*n* = 15) are shown as light colored lines and bars. The 24 hours light-dark cycle with 12 hours of light (inactive phase) and 12 hours of darkness (active phase) are indicated in different shadings behind the graphs. Boxplot represents median and quartiles with minimum and maximum whiskers; line graph presents mean ± SEM. ^*^P < 0.05, ^**^P < 0.01, ^***^P < 0.001.

Next, we tested if Cofilin inhibition in the sensory cortex changed the duration of social contact with conspecifics. We found a significant interaction between time and intervention group for social contact [*F*_(51.92, 4,886.54)_ = 20.14; *P* < 0.001]. The modeled longitudinal data showed that Cofilin suppression decreased the duration of social contact during two time points while it increased social contact during another time point (Fig. 6A, differences were found at hours 58.33, 76.67, and 120.00 to 121.67). Further analyses of the 12 h light and 12 h dark phases (the habitual inactive and active phases of this nocturnal species, respectively) showed that Cofilin suppression significantly decreased the expression of social contact during the active phases while this was unchanged during the inactive phases (Fig. 6B–D; active phase *P* < 0.001). In summary, these data indicated that suppression of Cofilin in the somatosensory cortex leads to functional changes in social contact selectively during the active phase.

### Overexpression of inactive Cofilin in the somatosensory cortex increases nest-hiding behavior

**Fig. 7:**
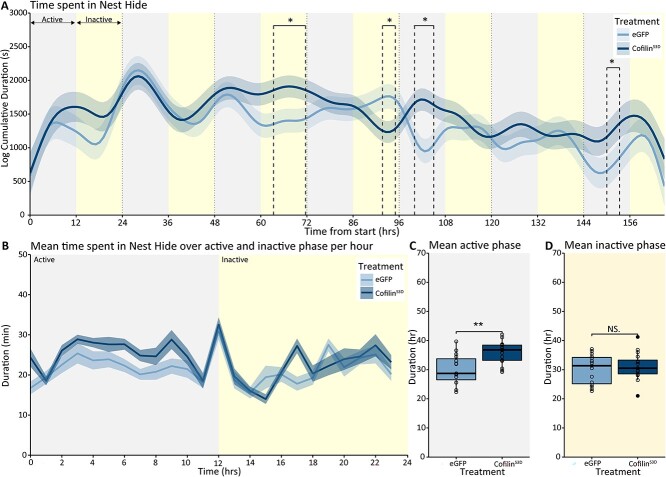
Nest hide behavior in BTBR Cofilin^S3D^ and control eGFP mice. Predicted time spent hiding in the nests during the 7 days of recording based on modeled data. A) Data are presented as the logarithm of cumulative duration (mean ± SEM) based on 1 h bins, with time from the start of the experiment on the *x*-axis in hours. B) Mean time spent hiding in the nests over the active and inactive phase of the recording averaged per hour of the active and inactive phase. Mean time spent hiding in the nests during the active C) and inactive D) phases over the recording period. BTBR-Cofilin^S3D^ animals (*n* = 16) are shown in dark colored lines and bars, and BTBR-eGFP controls (*n* = 15) are shown as light colored lines and bars. The 24 hours light-dark cycle with 12 hours of light (inactive phase) and 12 hours of darkness (active phase) are indicated in different shadings behind the graphs. Boxplot represents median and quartiles with minimum and maximum whiskers; line graph presents mean ± SEM. ^*^P < 0.05, ^**^P < 0.01, ^***^P < 0.001.

**Fig. 8:**
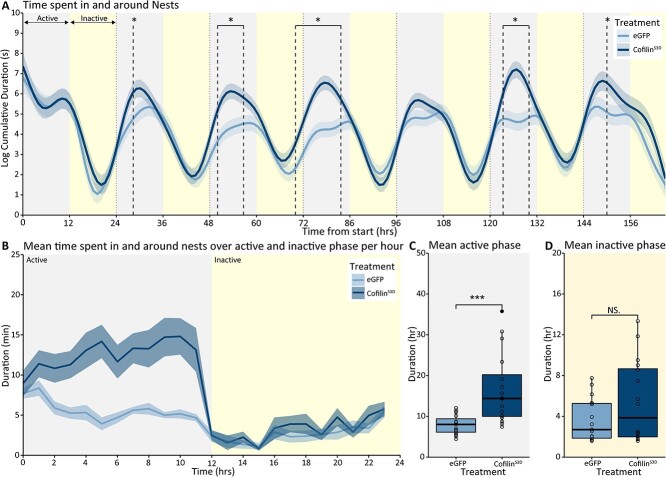
Time spent in and around the nests in BTBR Cofilin^S3D^ and control eGFP mice. Predicted time spent in and around the nests during the 7 days of recording based on modeled data. A) Data are presented as the logarithm of cumulative duration (mean ± SEM) based on 1 h bins, with time from the start of the experiment on the *x*-axis in hours. B) Mean time spent in and around the nests over the active and inactive phase of the recording averaged per hour of the active and inactive phase. Mean time spent in and around the nests during the active C) and inactive D) phase over the recording period. BTBR-Cofilin^S3D^ animals (*n* = 16) are shown in dark colored lines and bars, and BTBR controls (*n* = 15) are shown as light colored lines and bars. The 24 hours light-dark cycle with 12 hours of light (inactive phase) and 12 hours of darkness (active phase) are indicated in different shadings behind the graphs. Boxplot represents median and quartiles with minimum and maximum whiskers; line graph presents mean ± SEM. ^*^P < 0.05, ^**^P < 0.01, ^***^P < 0.001.

Subsequently, we investigated nest-hiding behavior after Cofilin suppression. BTBR-Cofilin^S3D^-treated mice spent more time hiding in the nests (treatment*time interaction: *F*_(24.71, 4,925.43)_ = 5.21; *P* < 0.001). Upon further inspection, the difference plots revealed a significant increase specifically during three time windows (61.6 to 70; 100 to 105; 150 to 153.3 h), while we observed a decrease during another brief time window (91.6 to 93.3) (Fig. 7A). However, overall, suppressing Cofilin activity increased the duration of nest hide during the active phase but not during the inactive phase (Fig. 7B and C; active phase *P* < 0.01). In addition to nest hiding, BTBR mice expressing Cofilin^S3D^ also showed increased behavioral activity levels in and around the nest boxes (treatment*time interaction: *F*_(33.69, 4,913.69)_ = 35.46; *P* < 0.001; Fig. 8A). Again, this increase in the time spent in and around the nest boxes was specifically present during the active phase (Fig. 8B−D; active phase *P* < 0.001). This observation shows that suppression of Cofilin leads to increased time spent both in and around the next boxes during the active phase.

### Overexpression of inactive Cofilin in the somatosensory cortex does not lead to overall differences in social approach, social leave, social sniff, and social follow

Altogether, Cofilin suppression alters social behavior in the BTBR, as observed by less time spent in social contact and in activity in and around the nest boxes in the BARISTA. Social contact is defined as interaction with < 2 cm distance between the conspecifics which includes behaviors such as social sniffing or social approach. Therefore, as a next step, we investigated further if these changes originate from alterations in specific aspects of social behavior such as social sniff, approach, or leave. Although some time points revealed specific decreases or increases, the overall mean duration of approach over the cumulative active or inactive phase did not differ between the Cofilin and eGFP-treated groups (Supplementary Fig. 4). Further analyses revealed that Cofilin suppression did not lead to overall changes in social sniff, social leave, or social follow (Supplementary Figs. 5−7). In summary, Cofilin inhibition does not change the overall time spent in social approach, social leave, social sniff, or social follow.

### Long bouts of social contact during the active phase are specifically decreased in Cofilin-treated mice

**Fig. 9:**
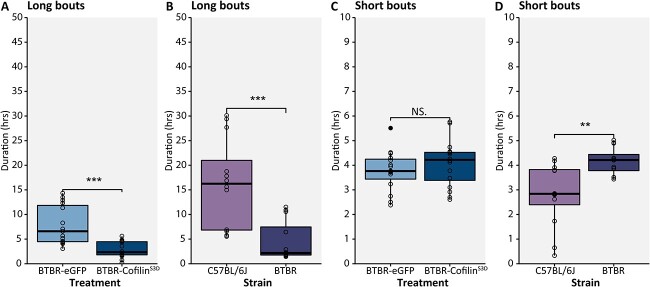
Long and short bouts of time spent in social contact in BTBR-eGFP vs CofilinS3D treated mice and strain differences between BTBR and C57BL6/J mice. Mean time mice spent in long bouts of social contact (>700 s per hour) during the active phase of the recording in A) BTBR-eGFP, and BTBR-Cofilin^S3D^ treated mice and B) C57BL/6J and BTBR mice. Mean time mice spent in short bouts of social contact (<700 s per hour) during the active phase of the recording in C) BTBR-eGFP, and BTBR-Cofilin^S3D^ treated mice and D) C57BL/6J, and BTBR mice. C57BL/6J: *n* = 12; BTBR: *n* = 12; BTBR-eGFP: *n* = 15; BTBR-Cofilin^S3D^: *n* = 16. Boxplot represents median and quartiles with minimum and maximum whiskers.^**^P < 0.01, ^***^P < 0.001.

To further investigate the decrease in social contact after Cofilin suppression, we assessed short and longer bouts of social contact. While social contact behaviors such as social sniffing are usually present in short bouts of contact, other behaviors such as huddling consist of longer bouts of social contact (Bove et al. 2018). Therefore, we performed an exploratory analysis in which social contact was divided into long and short bouts of social contact, defined as > or <700 s of social contact per hour during the active phase. This cut-off was determined by the assessment of peaks in social contact per hour as well as observation of huddling behavior in the recordings. Cofilin suppression did not alter the short bouts of social contact which corresponds with no changes found in social sniff, leave, follow, or approach (Fig. 9C). In contrast, Cofilin suppression decreased the longer bouts of social contact (Fig. 9A, *P* < 0.001).

This finding of decreased long bouts of social contact suggests that the difference in social contact mainly originates from these long bouts, possibly reflecting behaviors such as huddling. Since nest-hiding behaviors are increased during the active phase, this result again confirms that instead of spending long bouts in social contact in the open arena, Cofilin-treated mice spent more time in and around the nests. Interestingly, similar to the effects of Cofilin suppression, BTBR mice also spent less time in longer bouts of social contact during the inactive phase, when investigating strain differences between BTBR and C57BL/6J mice (Fig. 9B and D). Therefore, Cofilin suppression in the somatosensory cortex seems to exacerbate certain behavioral phenotypes that we observed in BTBR mice.

### Cofilin suppression in the somatosensory cortex does not alter social preference, social discrimination, or stereotyped behaviors

Subsequently, we wanted to identify if the changes in social contact in the BARISTA system could be mediated through changes in social preference or social discrimination. First, we carried out the social preference test to elucidate whether the BTBR mice prefer exploring a social stimulus over a nonsocial stimulus. Both BTBR groups preferred exploring the social stimulus and the social compartment (for all groups, *P* < 0. 001, Supplementary Fig. 8). This indicates that general sociability is intact in both groups. Secondly, we performed the direct social interaction task to assess short- and long-term social discrimination abilities. Preference for investigating the novel mouse indicates intact social discrimination abilities (Engelmann et al. 1995; Engelmann et al. 2011; Millan and Bales 2013). None of the groups showed a preference above chance levels for the novel social stimulus, indicating that Cofilin suppression does not restore general social discrimination deficits in the BTBR mice that have previously been found in BTBR mice (Supplementary Fig. 9) (Moy et al. 2007; Cope et al. 2022). In addition, we found no difference in the time mice spent in stereotyped exploratory patterns, novelty-induced hyperactivity, or in the expression of repetitive behaviors such as grooming, digging, rearing, or jumping (Supplementary Figs. 10 and 11). Together, these findings suggest that Cofilin suppression in the somatosensory cortex does not change, social preference, social discrimination, and stereotyped or repetitive behaviors.

**Fig. 10:**
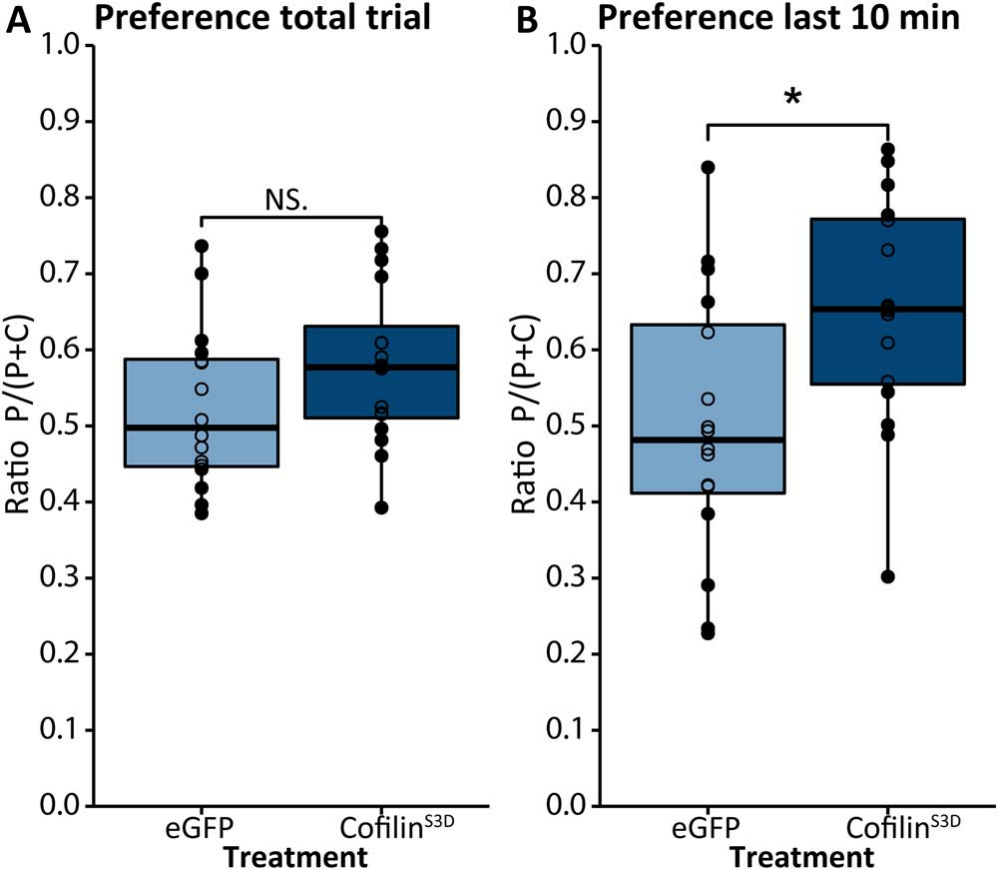
Preference for a tactile bedding stimulus after suppression of Cofilin. A) preference ratio for one type of bedding over the total 30 min trial. B) Preference for one type of bedding during the last 10 min of the trial. P = paperchip bedding and C = corncob bedding. BTBR- eGFP: *n* = 15; BTBR-Cofilin^S3D^: *n* = 16. Boxplot represents median and quartiles with minimum and maximum whiskers. ^*^P < 0.05.

### Altered time-dependent sensory bedding preference after Cofilin suppression

Lastly, we assessed the effect of Cofilin suppression on sensory phenotypes. We first performed the buried food test to assess the mouse’s general olfactory ability by testing the mouse’s ability to smell and uncover a food pellet. Cofilin suppression did not lead to any changes in performance, indicating that general olfactory ability is not affected by Cofilin suppression (Supplementary Fig. 12). Next, as a measure of tactile sensitivity, we performed a bedding preference task in which the mouse’s preference for exploring two types of bedding in the three-chamber apparatus is assessed. We observed no preference for one type of bedding over the complete trial in either experimental group (Fig. 10A). To see if the preference for a certain type of bedding was affected by novelty, we performed an exploratory analysis of the first, middle, and last 10 min. This analysis showed a significant main effect of both bedding type and time on exploration behavior (bedding: *F*(1,372) = 6.728, *P* < 0.01, time: *F*(2,372) = 8.464 m *P* < 0.001). A post hoc Tukey test revealed that in the last 10 min of the trial, Cofilin^S3D^-treated mice spent more time in the chamber containing paper chip bedding in comparison to the corncob-containing chamber (Fig. 10B and Supplementary Fig. 13, *P* = 0.02). This indicates that after initial exploration, BTBR-Cofilin^S3D^-treated mice prefer one type of bedding whereas control mice do not. Altogether, these observations indicate that Cofilin in the somatosensory cortex plays a specific role in tactile sensory processing.

## Discussion

In this study, we demonstrate, for the first time, a functional relationship between the somatosensory cortex and social behavior, mediated by the actin-regulating protein Cofilin. Suppressing Cofilin function in the somatosensory cortex of the BTBR mice leads to decreased social contact during longitudinal behavioral monitoring in the BARISTA-system. Instead, Cofilin-treated mice spent more time in and around the nest boxes. In addition to social behavior, Cofilin suppression also altered tactile preferences. Therefore, Cofilin regulation in the somatosensory cortex plays an important role in social functioning, possibly due to specific changes in tactile information processing.

Similar to our findings in BTBR mice, misregulation in Cofilin activity has also been found in gene knockout models for ASD, including the *Shank3* and *Ngln1* knockout models (Duffney et al. 2015; Liu et al. 2016). Moreover, Duffney et al. demonstrated that systemic inhibition of Cofilin activity in the *Shank3* mouse model leads to long-lasting rescues of social interaction deficits and repetitive behaviors. Specifically, local injection of a Cofilin inhibitor in the prefrontal cortex led to a rescue of social behavior. Here, we show that brain region-specific suppression of Cofilin in the somatosensory cortex also leads to changes in social behavior. In the present study, behavioral investigation revealed no differences in repetitive behavior or stereotyped behavior after this brain region-specific Cofilin intervention. In addition, no changes in social avoidance or social discrimination were found. Taken together, our work suggests that while the somatosensory cortex is directly involved in shaping certain forms of social behavior (i.e. consolidation of social contact), other brain areas such as the prefrontal cortex or striatum might be more heavily involved in the regulation of other forms of social behavior as well as repetitive and stereotyped behaviors. Indeed, after performing additional analysis of Cofilin levels in the striatum of BTBR and C57BL/6J mice, we also found increased Cofilin activity levels in the striatum of BTBR mice when compared to C57BL6/J controls (*P* = 0.035, C57BL/6J *n* = 5; BTBR *n* = 7). Therefore, we find changes in Cofilin regulation, indicating altered synaptic plasticity, in multiple brain regions involved in sensory processing and social behavior. This suggests that addressing the Cofilin misregulation in the somatosensory cortex as well as the striatum might be more effective in altering the sensory and social phenotypes in BTBR mice.

In addition to differences in target areas, an alternative reason why Cofilin inhibition in *Shank3* mice resulted in rescue of social deficits while in BTBR mice, the social contact phenotype was exacerbated after Cofilin manipulation could be related to experimental factors, such as differences in the used strain and method of Cofilin suppression. Here, we used a long-term local viral expression, while in the study of Duffney et al., Cofilin was inhibited either systemically or through a short-term local injection. In addition, our social behavioral outcome measures were measured in the BARISTA group housing system allowing for longitudinal individual behaviors in group-housed animals. While social behavior is classically tested using novel stimulus mice (Peça et al. 2011; Kouser et al. 2013; Zhou et al. 2016), we tested social behavior in groups of mice that were allowed to familiarize with each other during the weeks recording and therefore, our results did not depend on social novelty responses. Together, our results show altered social contact in BTBR mice after Cofilin suppression in the somatosensory cortex.

It is important to note that the observed decrease in social contact as a consequence of Cofilin suppression in the somatosensory cortex might be related to changes in tactile sensory behavior. In autistic people, it was shown that sensory-seeking behaviors correlate strongly to social and nonverbal communication impairments, and tactile hyporesponsivity correlates strongly with social and communication impairments (Foss-Feig et al. 2012). We found increased bedding preference in Cofilin-treated mice in a time-dependent manner. This was not an effect of novelty, as a preference was clearly seen during the last 10 min of the test. The question remains if the increase in preference after Cofilin suppression was an effect of differences in sensory discrimination abilities or in motivation to explore the sensory stimulus. This question needs to be addressed using further sensory behavioral testing, for example, by tactile discrimination task in head fixed mice after Cofilin manipulation or the olfactory habituation–dishabituation test. Furthermore, the exploration of bedding material relies on both tactile and olfactory cues. Since olfaction was unaffected in the buried food test, the difference in bedding preference is likely caused by differences in somatosensory processing. This indicates that Cofilin suppression in the somatosensory cortex affects both somatosensory processing and social behavior.

The decreased social contact after suppression of Cofilin could be a consequence of changes in a variety of social behaviors. We found that only longer bouts of social contact were significantly decreased during the active phase. This is in line with our findings that show no general changes in social sniff, follow, leave, or approach, behaviors that are commonly present in the shorter bouts. Therefore, these longer bouts of social behavior may reflect periods of huddling with a conspecific in the open arena of the BARISTA system. This is supported by the fact that BTBR-Cofilin^S3D^ mice show higher levels of behavioral activity in and around the nest boxes. Therefore, we conclude that instead of spending longer bouts in social contact in the arena, BTBR-Cofilin^S3D^ mice spent more time in their nest boxes.

Previous research into BTBR and C57BL/6J strain differences in the social colony reported a decrease in the total frequency of huddling in the open arena in BTBR mice (Bove et al. 2018), but no differences were found in the duration of huddling. Huddling is considered a socially inactive behavior that depends on somatosensory cortex activity (Bove et al. 2018; Naskar et al. 2019). It has been shown that synaptogenesis in the somatosensory cortex during development is time-locked with the development of huddling behavior in mice (Naskar et al. 2019). Social factors such as dominance and sex influence the amount of huddling. In addition, it is influenced by ambient temperature and thermogenic needs and has an important thermoregulatory function (Gilbert et al. 2010). Therefore, manipulation in the somatosensory cortex might lead to changes in social-sensory behaviors such as huddling activity. This is in line with the observed changes in social contact as well as in tactile preference. Additional assessment of social behavior in colonies that include both the C57BL/6J and BTBR strain after Cofilin inhibition or eGFP control injection would give more insight into the impact of Cofilin manipulation on behavioral changes in the respective strains. Moreover, additional research should encompass female mice to examine the potential generalizability of Cofilin manipulation on social behavior in this specific group.

The strain comparisons between BTBR and C57BL/6J mice in the current study revealed a decrease in social contact and increased nest hide behaviors in BTBR mice. Interestingly, Cofilin suppression in the somatosensory cortex of BTBR mice further decreased the time spent in social contact and led to increased nest hide. This indicates that inhibiting Cofilin function in the somatosensory cortex of BTBR mice may exacerbate specific behavioral differences observed in BTBR mice. While the suppression of Cofilin function may be beneficial to make memory processes resilient to sleep deprivation by stabilizing and preventing the loss of dendritic spines (Havekes et al. 2016b), this structural stability might also restrict receptor or vesicle relocation to the synapse (Raven et al. 2023). Therefore, suppression of Cofilin function may not be beneficial in some cases. Indeed, studies investigating Cofilin’s role in long-term potentiation (LTP), a process by which synaptic connections become stronger with frequent activation, have shown that Cofilin activation is necessary during the initial period after LTP induction (Chen et al. 2007; Bosch et al. 2014). In addition, late-phase LTP (8 h), but not early-phase LTP (30 to 50 min) is dependent on actin polymerization and reorganization of actin filaments, a process that is facilitated by Cofilin (Fukazawa et al. 2003; Lamprecht and LeDoux 2004). Therefore, elevated Cofilin activity might thus be beneficial for synaptic plasticity by supporting the initial stages of information processing. Indeed, Cofilin overactivation impaired long-term spatial memory, but improved short-term memory formation (Havekes et al. 2016a; Raven et al. 2023).

Importantly, the observation that increasing Cofilin function exacerbates the phenotypes also suggests that the observed increase in endogenous Cofilin activity in the somatosensory cortex of BTBR mice, compared to C57BL/6J mice, might be a compensatory mechanism in BTBR mice rather than directly driving the observed phenotypes. The higher levels of active Cofilin could potentially act as a compensatory mechanism for disturbed signal transmission and disturbed synaptic plasticity. By creating increased structural plasticity and receptor trafficking, this disturbed signal transmission may be partly compensated.

Another explanation for the observed behavioral phenotypes after Cofilin suppression is that the Cofilin misregulation could be the result of dysregulation of an upstream target that regulates the Cofilin pathway. Deficits in Rac1/PAK/LIMK/Cofilin signaling lead to aberrant synaptic plasticity, as well as impaired behavioral functioning (Hayashi et al. 2004; Golden et al. 2013). Therefore, targeting these upstream mechanisms might be more beneficial for altering synaptic plasticity than specifically altering Cofilin activity. For example, haploinsufficiency of an upstream regulator of Cofilin, PAK2, results in reduced Cofilin phosphorylation and impairments in the cytoskeleton of the synapse as well as decreased *Shank3* expression in the brain (Wang et al. 2018). Furthermore, this haploinsufficiency also led to autism-like behavior (Wang et al. 2018). Moreover, in *Shank3* knockout mice, social deficits are rescued by inhibiting Cofilin or activating Rac1, an upstream Cofilin regulator (Duffney et al. 2015). Reversely, in wild-type mice, social deficits can be induced by inhibiting PAK or Rac1 (Duffney et al. 2015). Together, these findings indicate that upstream interventions of the Rac1/PAK/LIMK/Cofilin signaling pathway might have more widespread effects on behavioral and molecular mechanisms. However, it is unclear if similar upstream molecular changes mediate the behavioral differences found in this study. Therefore, further research on spine profiles and upstream synaptic proteins is needed to elucidate which molecular changes mediate the observed behavioral differences described in this thesis.

In summary, this study demonstrates that targeting structural plasticity through manipulation of Cofilin in the somatosensory cortex leads to changes in social behavior and tactile sensory behavior in BTBR mice. Our results, therefore, show a functional relationship between the somatosensory cortex and social functioning through processes involved in synaptic adaptation. Previous studies have put forward Cofilin regulation as a potential pathophysiological mechanism underlying multiple neurological disorders including ASD in which Cofilin is dysregulated (Duffney et al. 2015; Liu et al. 2016; Shaw and Bamburg 2017). The results here bring forward Cofilin as one of the potential neurobiological substrates involved in the functional relationship between sensory and social functioning. Together, these findings highlight the relevance of synaptic plasticity as an important process in the expression of behavior in autistic people.

## Supplementary Material

Supplementary_Riemersma_et_al_2023_Manuscript_Cofilin_BTBR_revised_bbae136
