# Supplementary material for: Suppression of Cofilin function in the somatosensory cortex alters social contact behavior in the BTBR mouse inbred line
Source: Cereb Cortex. 2024 Apr 10;34(4):bhae136. doi: 10.1093/cercor/bhae136 (PMC11008688; doi:10.1093/cercor/bhae136)
Supplement: Supplementary_Riemersma_et_al_2023_Manuscript_Cofilin_BTBR_revised_bbae136 [file supplementary_riemersma_et_al_2023_manuscript_cofilin_btbr_revised_bbae136.docx]

**SUPPLEMENTARY INFORMATION**

**Suppression of Cofilin function in the somatosensory cortex alters social contact behavior in the BTBR mouse inbred line**

**Riemersma *et al.***

**Supplementary Table 1 Overview of the GAMM models used for BARISTA analysis of BTBR and C57BL/6 strain differences**

| **Behavior in BARISTA** | **Model** |
| --- | --- |
| Social contact duration (log transformed) | ~ s(time, by=strain, k=40) + strain + s(id, bs="fs", m=1) + s(time, Colony, bs="fs", m=1) |
| Distance moved duration | ~ s(time, by=strain, k=80) + strain + s(time, id, bs="fs", m=1) + s(time, Colony, bs="fs", m=1), family=quasipoisson()) |
| Nest Hide duration | ~ s(time, by=strain, k=40) + strain + s(time, id, bs="fs", m=1) + s(time, Colony, by=strain, bs="fs", m=1), family=quasipoisson()) |
| Contact in and around nests | ~ s(time, by=strain, k=40) + strain + s(time, id, bs="fs", m=1) + s(time, Colony, by=strain, bs="fs", m=1), family=quasipoisson()) |

**Supplementary Table 2 Overview of the GAMM models used for BARISTA analysis of BTBR-CofilinS3D and BTBR-eGFP mice**

| **Behavior in BARISTA** | **Model** |
| --- | --- |
| Social contact duration (log transformed) | ~ s(time, by=treatment, k=80) + treatment + s(time, id, bs="fs", m=1) + s(time, Colony, bs="fs", m=1) |
| Distance moved duration | ~ s(time, by=treatment, k=80) + treatment + s(time, id, bs="fs", m=1) + s(time, Colony, bs="fs", m=1), family=quasipoisson() |
| Nest Hide duration (X^1/2 transformed) | ~ s(time, by=treatment, k=40) + treatment + s(time, id, bs="fs", m=1) + s(time, Colony, bs="fs", m=1) |
| Social Leave duration (log transformed) | ~ s(time, by=treatment, k=40) + treatment + s(id, bs="fs", m=1) + s(time, Colony, bs="fs", m=1), |
| leave frequency (log transformed) | ~ s(time, by=treatment, k=40) + treatment + s(id, bs="fs", m=1) + s(time, Colony, bs="fs", m=1) |
| Social contact frequency | ~ s(time, by=treatment, k=80) + treatment + s(time, id, bs="fs", m=1) + s(time, Colony, by=treatment, bs="fs", m=1), family=quasipoisson() |
| Contact in and around nests duration (log transformed) | ~ s(time, by=treatment, k=40) + treatment + s(time, id, bs="fs", m=1) + s(time, Colony, bs="fs", m=1) |
| Social sniff duration (log transformed) | ~ s(time, by=treatment, k=80) + treatment + s(time, id, by=treatment, bs="fs", m=1) + s(time, Colony, bs="fs", m=1) |
| Social sniff frequency (log transformed) | ~ s(time, by=treatment, k=40) + treatment + s(time, id, bs="fs", m=1) + s(time, Colony, bs="fs", m=1) |
| Social approach duration (log transformed) | ~ s(time, by=treatment, k=80) + treatment + s(time, id, bs="fs", m=1) + s(time, Colony, bs="fs", m=1), |
| Passive approach duration (log transformed) | ~ s(time, by=treatment, k=80) + treatment + s(time, id, bs="fs", m=1) + s(time, Colony, bs="fs", m=1) |
| Social approach frequency (log transformed) | ~ s(time, by=treatment, k=80) + treatment + s(time, id, bs="fs", m=1) + s(time, Colony, bs="fs", m=1) |
| Social Follow duration (log transformed) | ~ s(time, by=treatment, k=80) + treatment + s(time, id, bs="fs", m=1) + s(time, Colony, bs="fs", m=1) |
| Passive social follow duration (log transformed) | ~ s(time, by=treatment, k=80) + treatment + s(time, id, bs="fs", m=1) + s(time, Colony, bs="fs", m=1) |


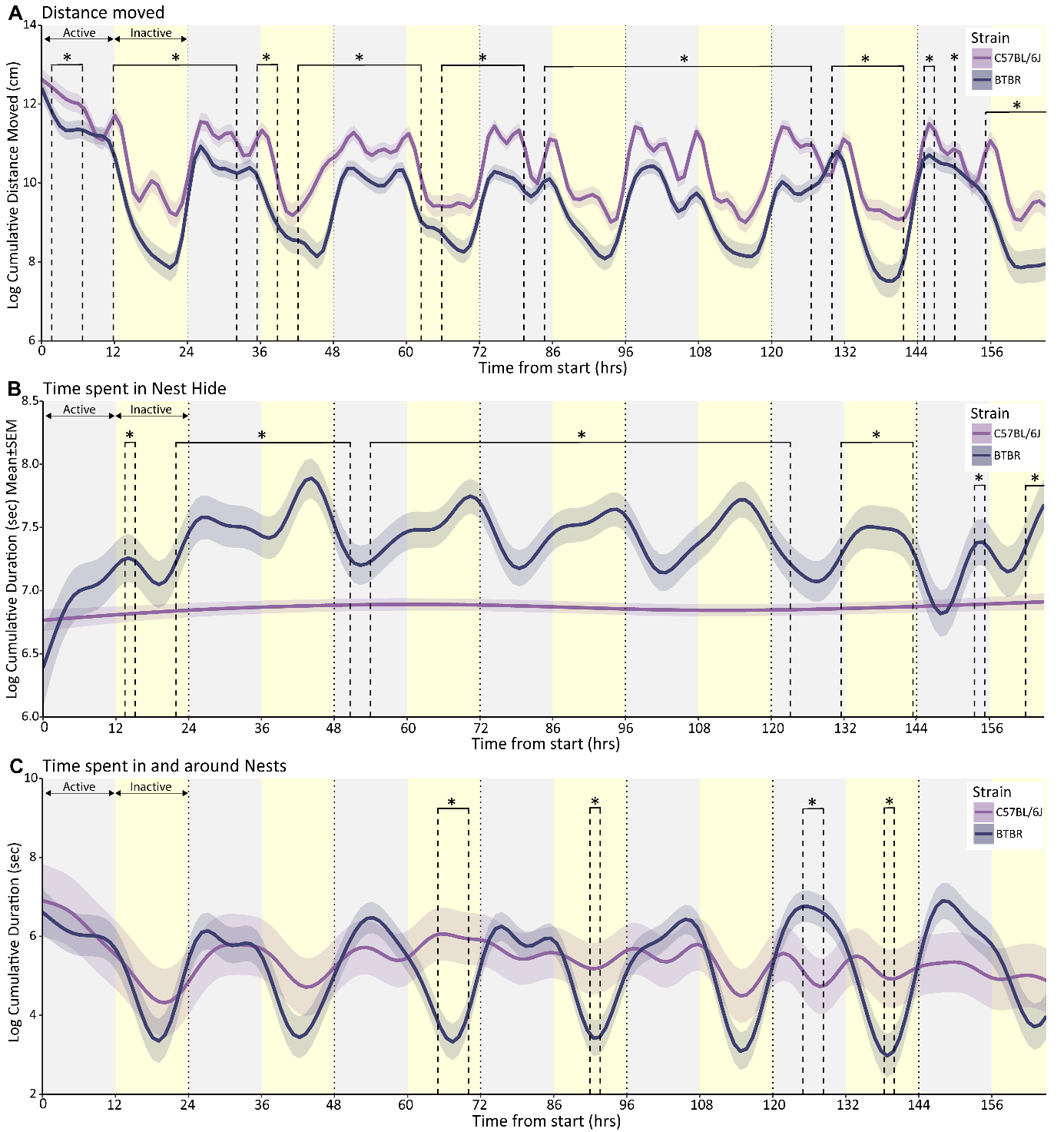


**Supplementary Figure 1. Strain differences in locomotor activity and nest hiding behaviors between BTBR and C57BL/6J mice in BTBR Cofilin^S3D^ and control eGFP mice.** Predicted time spent in distance moved, **A**, time spent in nest hide, **B**, and time spent in and around the Nests, **C**, during the seven days of recording in the BARISTA system based on modeled data. Data is presented as the logarithm of cumulative duration (mean±SEM) based on 1-hour bins, with time from the start of the experiment on the x-axis in hrs. BTBR animals (N=12) are shown in dark purple, and C57BL/6J controls (N=12) are shown in light purple. Yellow shading behind the graph indicates the inactive phase, whereas grey shading indicates the active phase. The line graph presents mean ± SEM. *p<0.05, **p<0.01, ***p<0.001

**
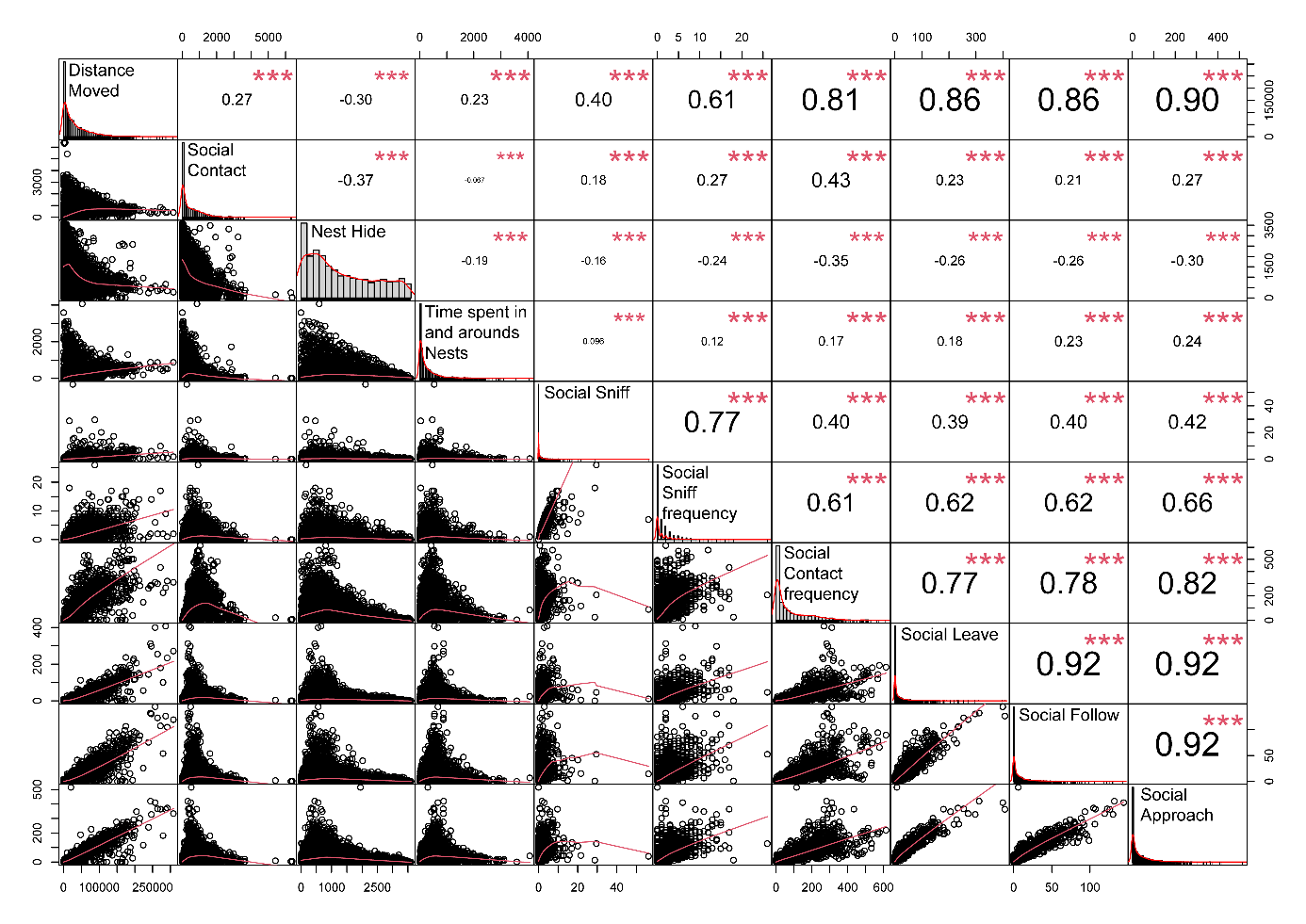
**

**Supplementary Figure 2. Correlation Matrix of the outcome measure in the BARISTA system for BTBR and C57BL/6J mice.** Distance moved, social sniff frequency, social contact frequency, social leave duration, social follow duration and social approach are strongly correlated. Social contact duration, time spent in and around the nest boxes, nest hide, and social sniff are not strongly correlated with other outcome measures.


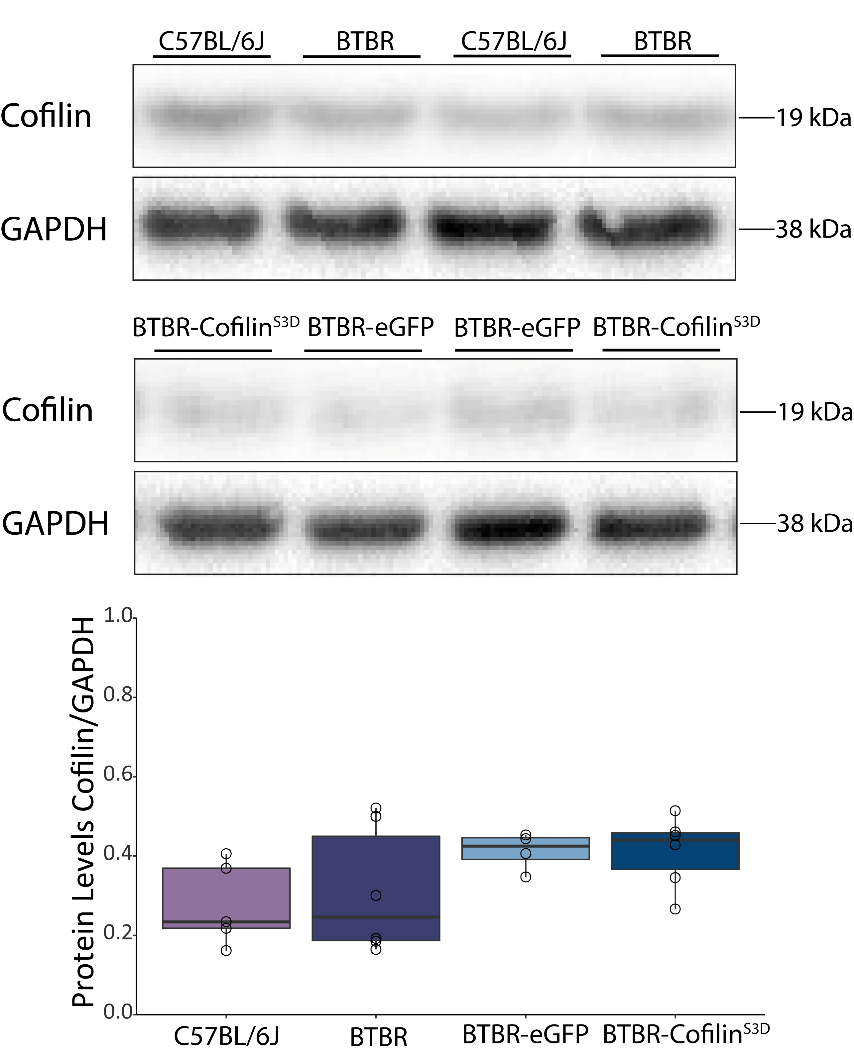


**Supplementary Figure 3. Total Cofilin levels in C57BL/6J, naïve BTBR, eGFP- and Cofilin^S3D^ injected mice.** Western blot analysis of total Cofilin (Cofilin) protein levels in the somatosensory cortex. GAPDH was used as an internal control, representative blots are shown. Each band represents an individual animal (p>0.05; C57BL/6J n=5; BTBR n=6; BTBR-eGFP n=4, BTBR-Cofilin^S3D^ n=6). Boxplot represents median and quartiles with minimum and maximum whiskers.


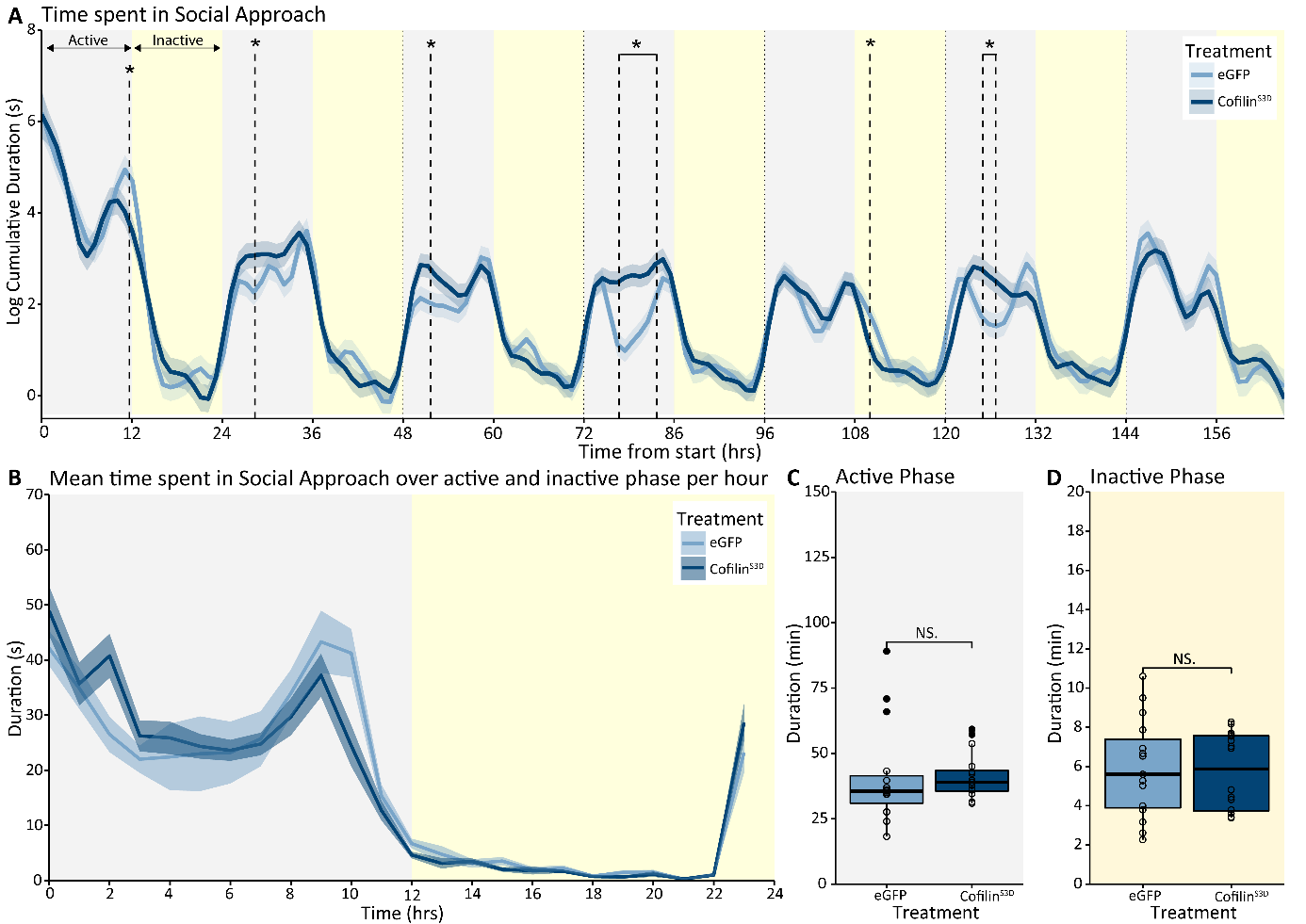


**Supplementary Figure 4. Time spent in Social Approach in BTBR Cofilin^S3D^ and control eGFP mice.** **A,** Predicted time spent in social approach during the seven days of recording in the BARISTA based on modeled data. Data is presented as the logarithm of cumulative duration (mean±SEM) based on 1-hour bins, with time from the start of the experiment on the x-axis in hrs. **B,** Mean time spent in social approach over the active and inactive phase of the recording per hour of the active and inactive phase. Mean time spent in social approach during the active, **C,** and inactive, **D,** phase over the recording period. BTBR- Cofilin^S3D^ animals (N=16) are shown in dark blue, and BTBR controls (N=15) are shown as light blue lines. Yellow shading behind the graph indicates the inactive phase, whereas grey shading indicates the active phase. Boxplot represents median and quartiles with minimum and maximum whiskers; line graph presents mean ± SEM. *p<0.05


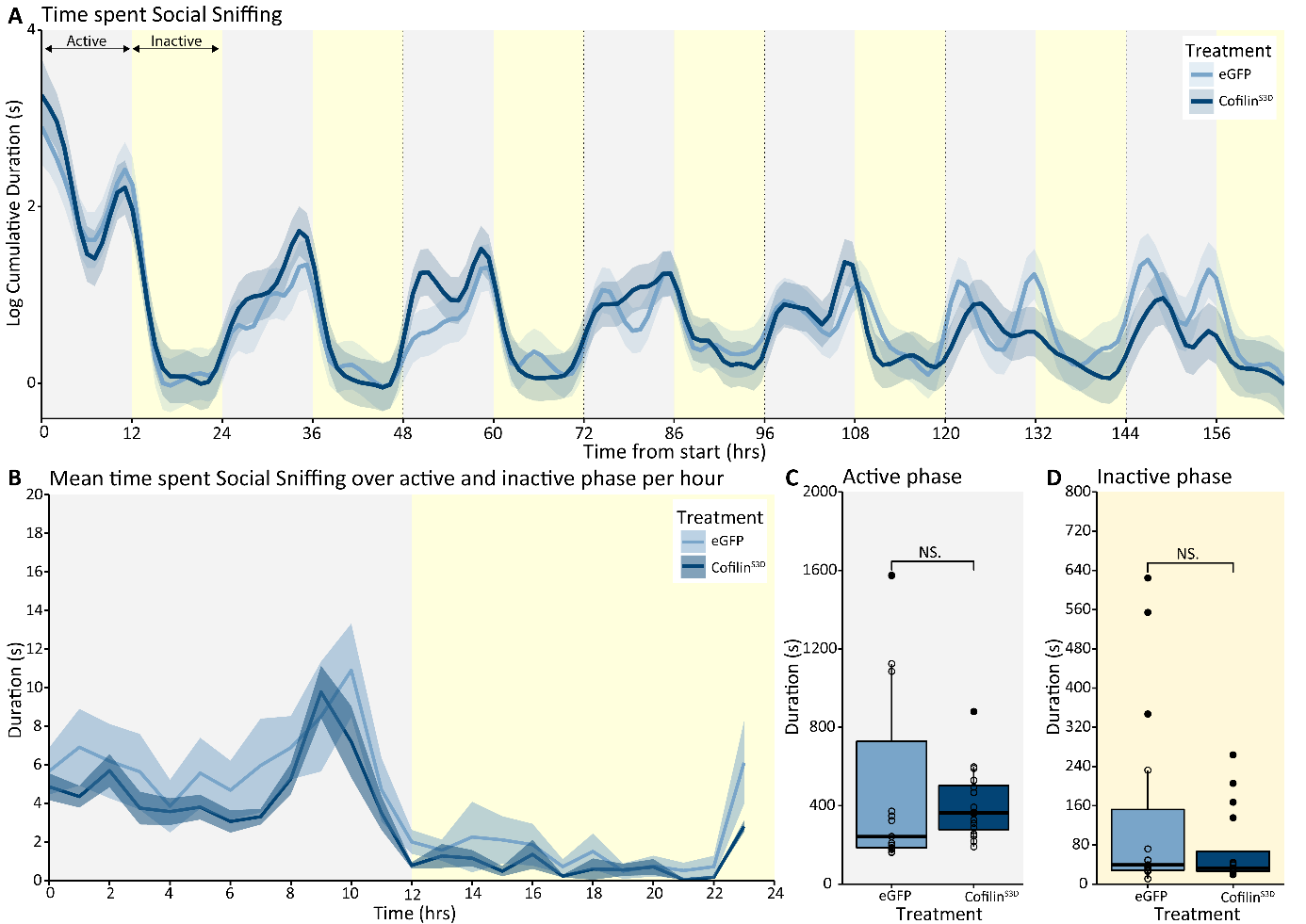


**Supplementary Figure 5. Time spent Social Sniffing in BTBR Cofilin^S3D^ and control eGFP mice.** **A,** Predicted time spent social sniffing during the seven days of recording in the BARISTA based on modeled data. Data is presented as the logarithm of cumulative duration (mean±SEM) based on 1-hour bins, with time from the start of the experiment on the x-axis in hrs. **B,** Mean time spent social sniffing over the active and inactive phase of the recording per hour of the active and inactive phase. Mean time spent social sniffing during the active, **C,** and inactive, **D,** phases over the recording period. BTBR- Cofilin^S3D^ animals (N=16) are shown in dark blue, and BTBR controls (N=15) are shown as light blue lines. Yellow shading behind the graph indicates the inactive phase, whereas grey shading indicates the active phase. Boxplot represents median and quartiles with minimum and maximum whiskers; line graph presents mean ± SEM. *p<0.05


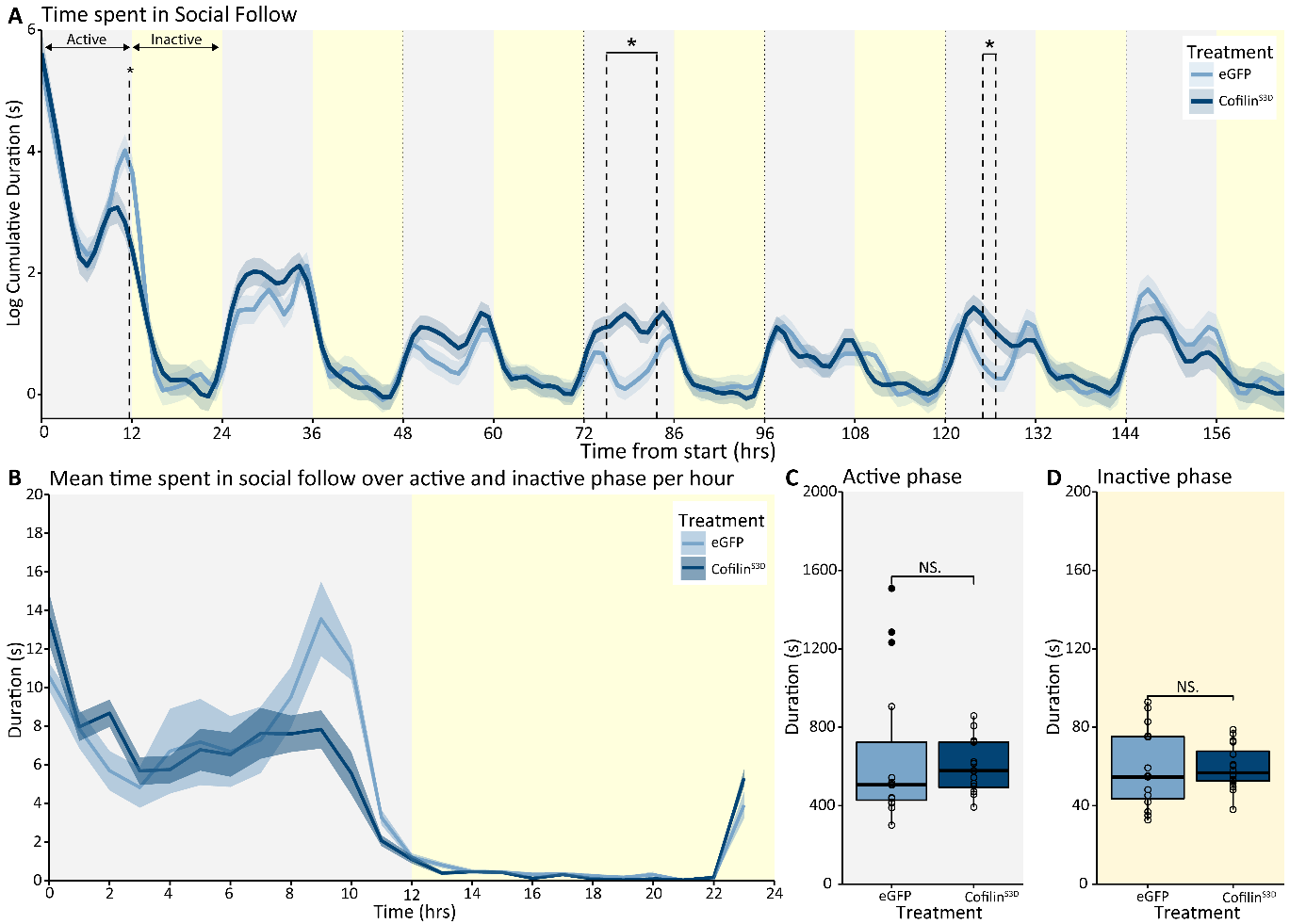


**Supplementary Figure 6. Time spent in Social Follow in BTBR Cofilin^S3D^ and control eGFP mice.** **A,** Predicted time spent in social follow during the seven days of recording in the BARISTA based on modeled data. Data is presented as the logarithm of cumulative duration (mean±SEM) based on 1-hour bins, with time from the start of the experiment on the x-axis in hrs. **B,** Mean time spent in social follow over the active and inactive phase of the recording per hour of the active and inactive phase (mean±SEM). Mean time spent in social follow during the active, **C,** and inactive, **D,** phase over the recording period. BTBR- Cofilin^S3D^ animals (N=16) are shown in dark blue, and BTBR controls (N=15) are shown as light blue lines. Yellow shading behind the graph indicates the inactive phase, whereas grey shading indicates the active phase. Boxplot represents median and quartiles with minimum and maximum whiskers; line graph presents mean ± SEM. *p<0.05


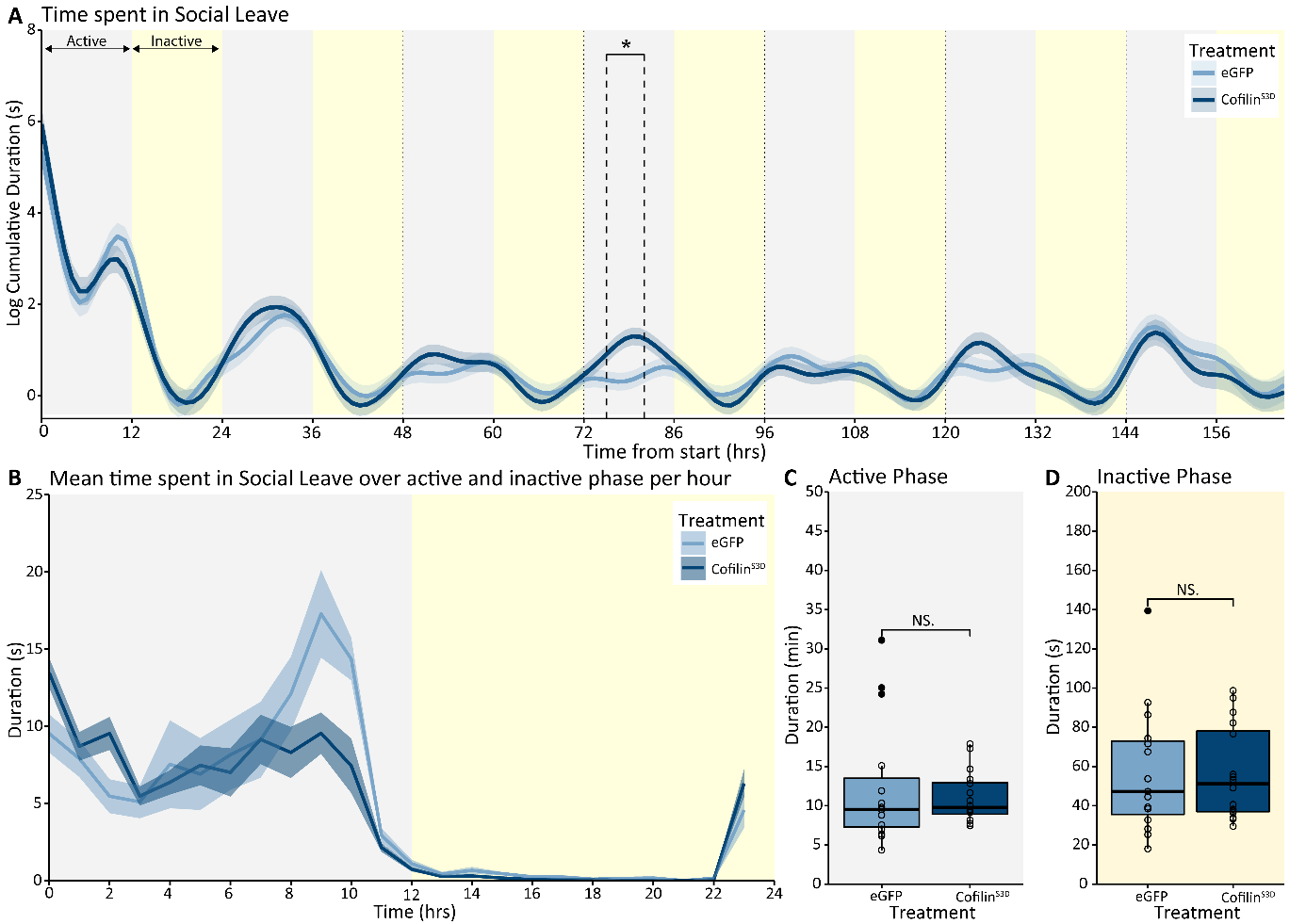


**Supplementary Figure 7. Time spent in Social Leave in BTBR Cofilin^S3D^ and control eGFP mice.** **A,** Predicted time spent in social leave during the seven days of recording in the BARISTA based on modeled data. Data is presented as the logarithm of cumulative duration (mean±SEM) based on 1-hour bins, with time from the start of the experiment on the x-axis in hrs. **B,** Mean time spent in social leave over the active and inactive phase of the recording per hour of the active and inactive phase (mean±SEM). Mean time spent in social leave during the active, **C,** and inactive, **D,** phases over the recording period. BTBR- Cofilin^S3D^ animals (N=16) are shown in dark blue, and BTBR controls (N=15) are shown as light blue lines. Yellow shading behind the graph indicates the inactive phase, whereas grey shading indicates the active phase. Boxplot represents median and quartiles with minimum and maximum whiskers; line graph presents mean ± SEM. *p<0.05


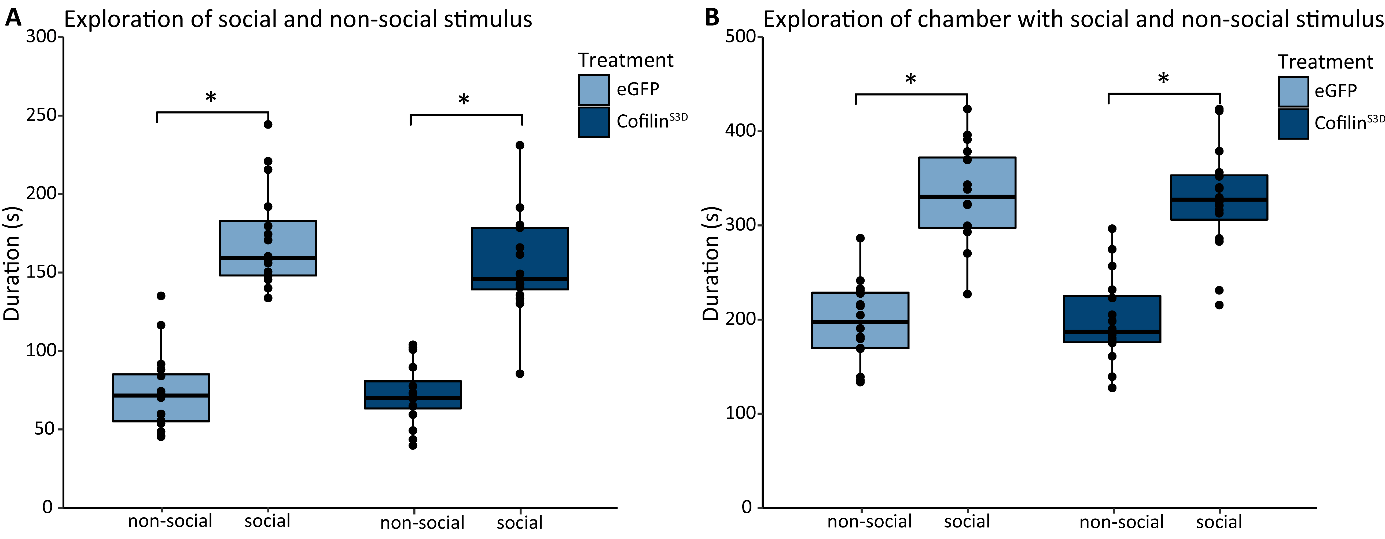


**Supplementary Figure 8. Social preference in BTBR Cofilin^S3D^ and control eGFP mice.** Mice were allowed to explore the three-chamber apparatus with in one chamber a cage with a social stimulus and in the other chamber an empty cage. Exploration time of the social stimulus and non-social stimulus, **A**, and the chamber with the social and non-social stimulus in the three-chamber social preference task, **B**, was assessed. BTBR- eGFP: N=15; BTBR-Cofilin^S3D^: N=16. Boxplot represents median and quartiles with minimum and maximum whiskers. *p<0.05


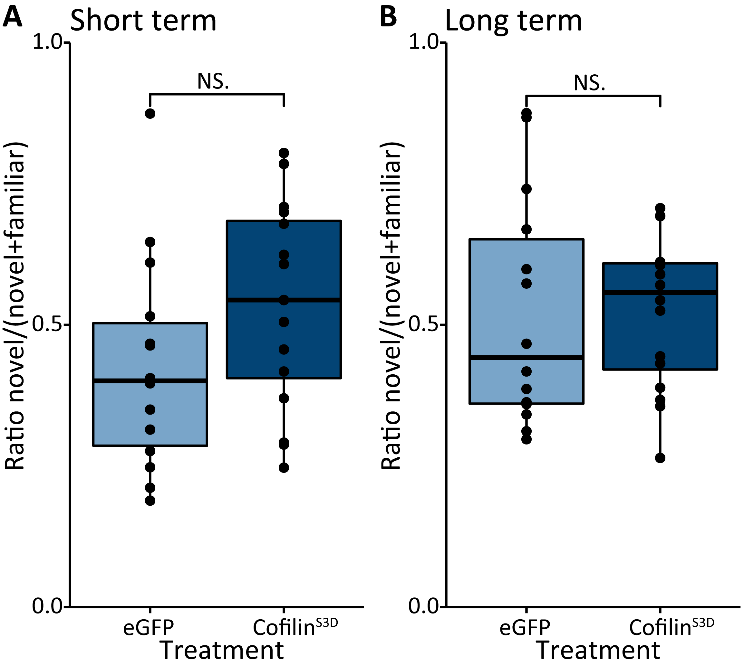


**Supplementary Figure 9. Social discrimination in BTBR Cofilin^S3D^ and control eGFP mice.** Mice were allowed to directly interact with an A/J stimulus animal in a normal housing cage. In the learning phase, animals were familiarized with one stimulus animals. After a 5-minute short term interval, **A,** and a 24-hour long term interval, **B,** the ability of the mice to recognize a novel over the familiar stimulus animal was assessed. BTBR- eGFP: N=15; BTBR-Cofilin^S3D^: N=16. Boxplot represents median and quartiles with minimum and maximum whiskers.


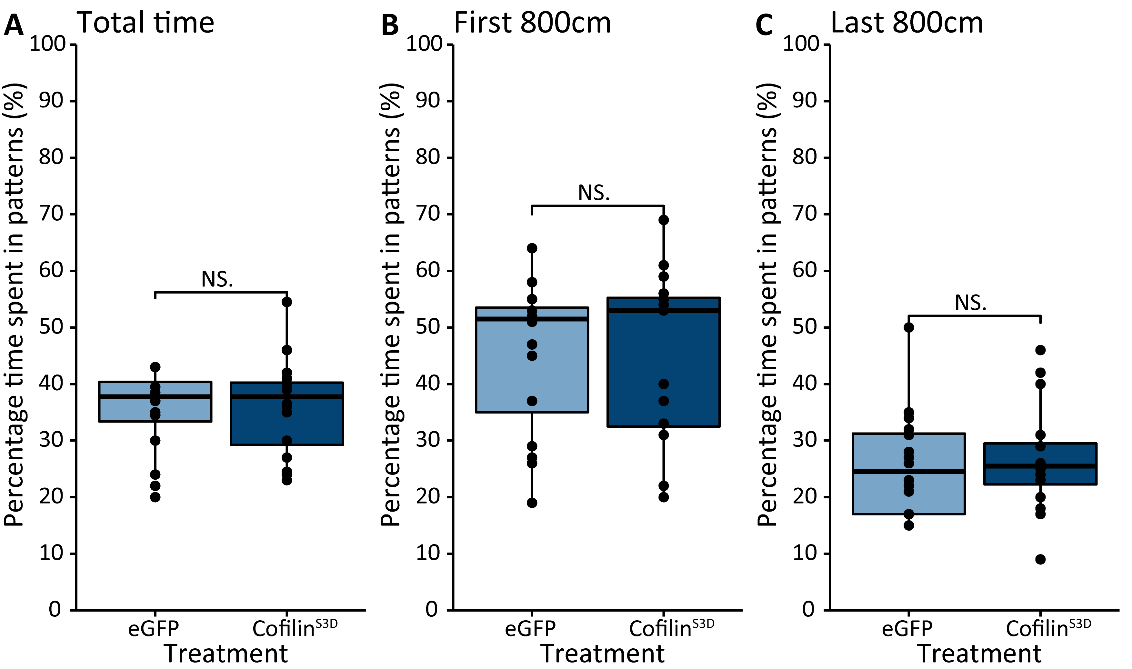


**Supplementary Figure 10. Repetitive exploratory patterns in BTBR Cofilin^S3D^ and control eGFP mice.** Repetitive exploratory patterns were analyzed after a one-hour exploration in a standard housing cage. No differences were found in the percentage of time spent in stereotyped exploratory patterns over the entire trial **A,** and during the first, **B**, and last, **C,** 800 cm of the trial to correct for potential differences in locomotor activity. BTBR- eGFP: N=15; BTBR-Cofilin^S3D^: N=16. Boxplot represents median and quartiles with minimum and maximum whiskers.


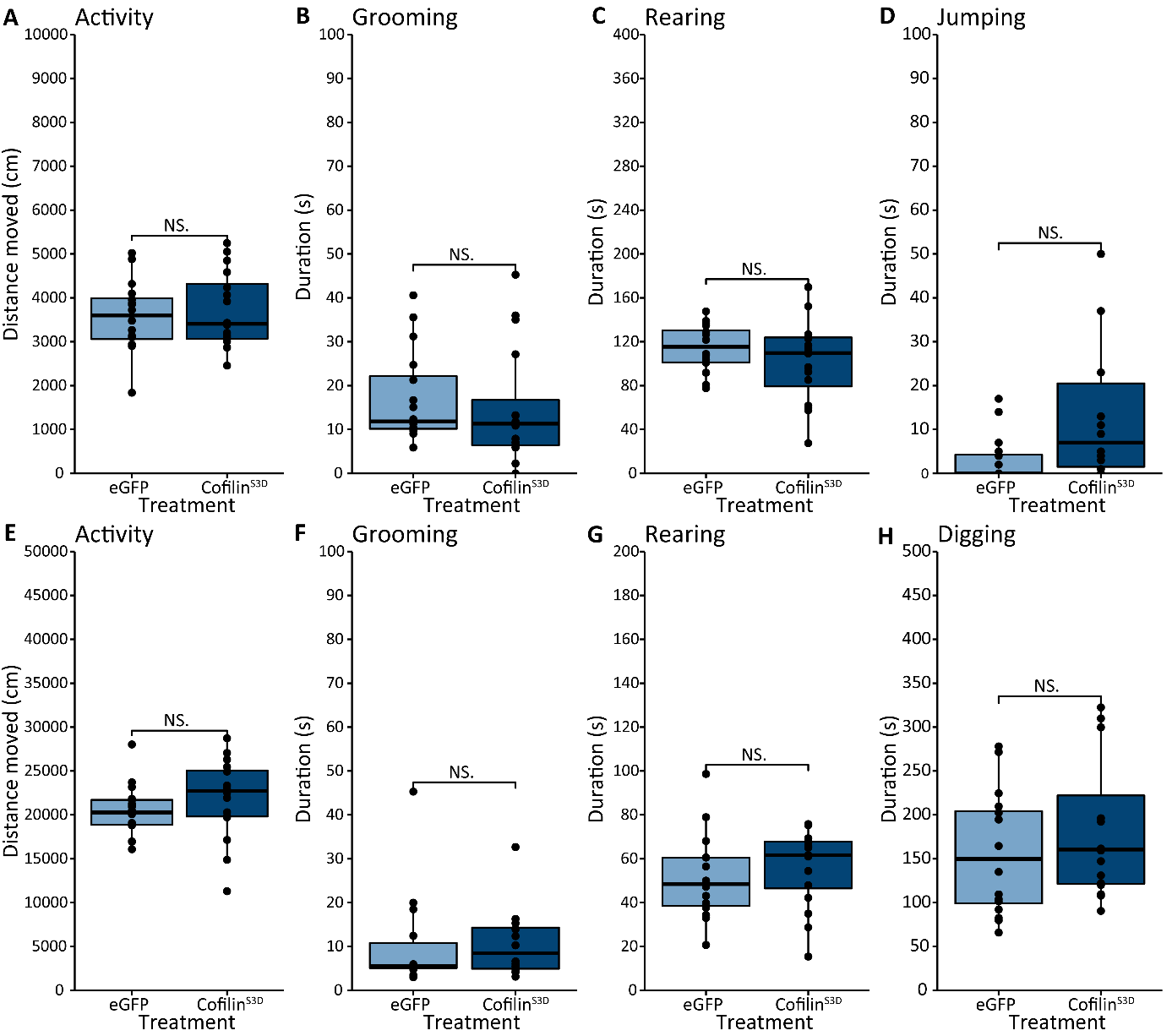


**Supplementary Figure 11. Repetitive behavior in BTBR Cofilin^S3D^ and control eGFP mice.** Spontaneous repetitive behavior was measured during the first 10 minutes of exploration in a standard housing cage without and with bedding. In cages without bedding locomotor activity, **A**, grooming, **B**, rearing, **C**, and jumping, **D**, was assessed. In cages with bedding locomotor activity, **E**, grooming, **F**, rearing, **G**, and digging, **H**, was assessed. BTBR- eGFP: N=15; BTBR-Cofilin^S3D^: N=16. Boxplot represents median and quartiles with minimum and maximum whiskers.


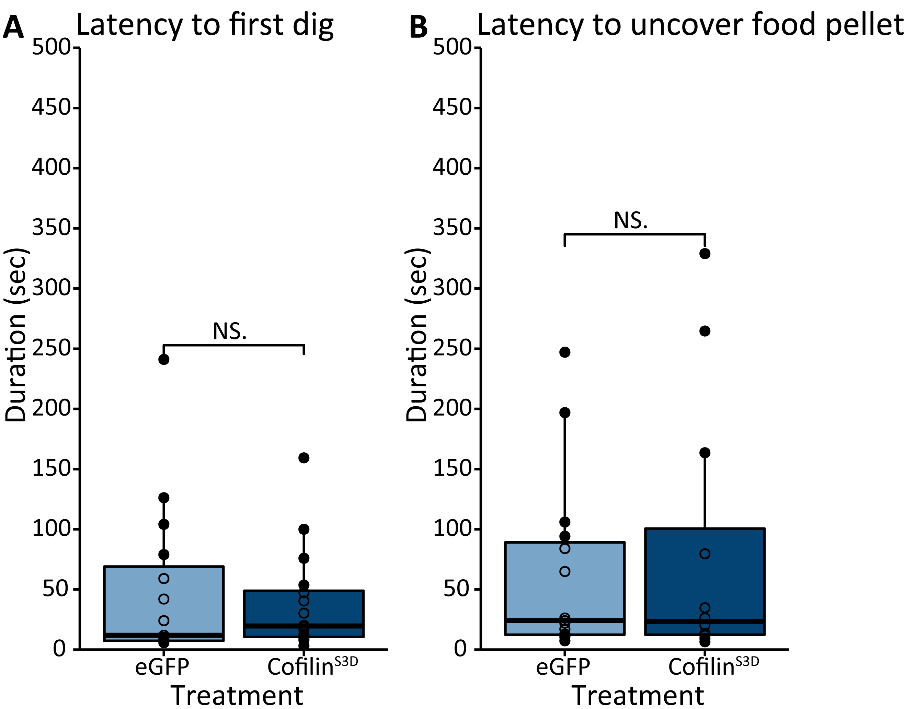


**Supplementary Figure 12. Latency to dig for a food pellet in the Buried food test in BTBR Cofilin^S3D^ and control eGFP mice. A.** Latency to the first dig at the right location of the food pellet. **B.** Latency to uncover the buried food pellet. BTBR- eGFP: N=15; BTBR-Cofilin^S3D^: N=16. Boxplot represents median and quartiles with minimum and maximum whiskers.


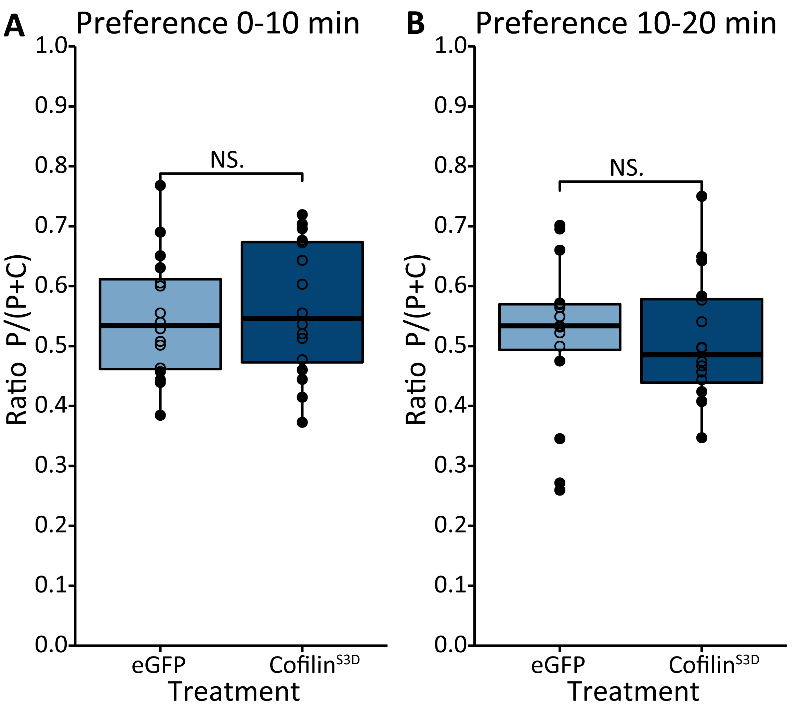


**Supplementary Figure 13. Bedding preference in BTBR Cofilin^S3D^ and control eGFP mice.** The preference for one type of bedding presented in the three-chamber apparatus during the first 10 minutes of the trial, **A,** and the middle 10 minutes, **B,** of the total 30-minute trial. Preference is presented as the ratio of exploration of the paperchip (P) bedding, divided by total exploration times of both types of bedding: paperchip (P) and corncob (C). BTBR- eGFP: N=15; BTBR-Cofilin^S3D^: N=16. Boxplot represents median and quartiles with minimum and maximum whiskers.
